# Diversity-Specific Empowering Leadership: An Alternative Approach to Reducing Sex-Based Bias and Enabling Inclusivity

**DOI:** 10.1007/s10551-025-05973-3

**Published:** 2025-03-11

**Authors:** Cara-Lynn Scheuer, Catherine Loughlin, Danielle Prowse, Corinne McNally, Kara A. Arnold, Shasanka Chalise

**Affiliations:** 1 Wall College of Business, Coastal Carolina University, P.O. Box 261954, Conway, SC 29528-6054 USA; 2 Faculty of Management, Dalhousie University, Rowe Building, 6100 University Avenue, PO Box 15000, Halifax, NS B3H 4R2 Canada; 3 Sobey School of Business, Saint Mary’s University, 923 Robie St., Halifax, NS B3H 3C3 Canada; 4 Faculty of Business Administration, Memorial University, St. John’s, NL A1B 3X5 Canada; 5 Hill & Levene Schools of Business, University of Regina, Regina, SK S4S 0A2 Canada

**Keywords:** Empowering leadership, Sex-based bias and stereotypes, Female leaders, Competence, Diversity beliefs, Climate for inclusion

## Abstract

Achieving sex-based equity in organizational leadership roles has proven to be a ‘wicked’ problem with existing diversity initiatives providing minimal improvement. In this paper, we address this issue by considering a key inhibiter to women’s leadership advancement—biased perceptions of female leaders’ competence—and links to a climate for inclusion. In Study 1 (*N* = 236), we develop and validate a Diversity-Specific Empowering Leadership (DSEL) measure, and demonstrate its value in predicting perceptions of female leaders’ competence when compared to alternative leadership models (empowering leadership, transformational leadership, diversity-specific transformational leadership, transactional leadership, leader diversity-valuing behavior, and inclusive leadership). In Study 2 (*N* = 314), we introduce sex-based diversity beliefs as a moderator in the relationship between DSEL and perceptions of female leaders’ competence. In Study 3 (*N* = 313), we provide support for a mediated moderation model, with sex-based diversity beliefs moderating the effects of DSEL on perceptions of female leaders’ competence. In turn, this is associated with a climate for inclusion. DSEL is collaborative and developmentally focused, and our findings suggest it may attenuate sex-based biases in perceptions of leadership, especially for those who have been most resistant to change (i.e., individuals with negative sex-based diversity beliefs). Our research offers theory that can support ethical action by advancing DSEL as a promising ‘target-specific’ leadership model for creating less biased and more inclusive work environments for all.

Women comprise nearly half of the labor force and research has demonstrated they are equally, and sometimes more, competent in leadership ability compared to men (e.g., Eagly et al., 1995; Gipson et al., 2017; Lyness & Heilman, 2006; Lemoine & Blum, 2021; McKinsey & Company, 2021a; Meyer et al., 2022; Nordell, 2021; Paustian-Underdahl et al., 2014; Sergent & Stajkovic, 2020). However, women remain severely underrepresented in leadership positions, holding only 6.0% of CEO positions in Standard & Poor’s 500, 7.3% in Fortune 1000 firms in the United States, and 4% in Canada's largest publicly traded companies (Canadian Women’s Foundation, 2022; Catalyst, 2021; Women Business Collaborative, 2021) with similar trends seen in other parts of the world (Chugh & Sahgal, 2007; McKinsey & Company, 2021b, c). Unfortunately, research suggests progress for women in leadership positions has not only stalled (Caleo & Heilman, 2019), it is reversing (Elsesser, 2022; The Verian Group, 2024). Plausible explanations for this include the continued effects of sex-based bias in organizations (Bernstein et al., 2020; Nordell, 2021), and the unintended negative consequences that can arise from organizational diversity initiatives due to backlash (Bernstein et al., 2020; Caleo & Heilman, 2019; Leslie, 2019).

From a stakeholder theory perspective, effective managerial practice involves inclusive decision-making (Bernstein et al., 2020), and the justice rationale (or moral motive) requires leaders “to establish equal access, fair treatment, and workplace environments that are free from discrimination and harassment” (Dover et al., 2020; p. 155). Further, based on the business case for diversity (Cox & Blake, 1991), organizations recognize they cannot be globally competitive if they recruit and retain leaders from only half of the workforce (McKinsey & Company, 2015, 2021a, b, c; Valerio & Sawyer, 2016). Nevertheless, reports show that organizations are failing to follow through on creating and sustaining diverse and inclusive work environments (Bernstein et al., 2020; Canadian Centre for Diversity & Inclusion, 2019; The Conference Board of Canada, 2013; McKinsey & Company, 2021a, b, c). Additionally, research suggests that many attempts to address diversity via organizational-level interventions do not have the desired impact (Caleo & Heilman, 2019; Dobbin & Kalev, 2018; Dover et al., 2020; Foley & Williamson, 2019; Leslie, 2019). According to a 2017 report, Fortune 500 companies collectively spend more than $16 billion annually on diversity management (Staley, 2017). Yet, research repeatedly demonstrates that these and other diversity initiatives often backfire (Caleo & Heilman, 2019), eliciting defensiveness from the very individuals this training is designed to impact (e.g., Chang et al., 2019a, b; Dover et al., 2020). Systematic reviews (Bezrukova et al., 2012; Kulik & Roberson, 2008a, b) and meta-analyses (Bezrukova et al., 2016) demonstrate that while diversity initiatives may have positive effects on immediate attitudes toward diversity and knowledge about others, their impact on prejudice and behaviors toward disadvantaged groups (e.g., perceptions of female leaders’ competence) are less successful (Dover et al., 2020). The effects of diversity initiatives have also been shown to diminish with time (Bezrukova et al., 2016). In summary, when it comes to improving the representation and inclusion of women at all levels of organizations there appears to be a gap between policy and performance (The Conference Board of Canada, 2013; Nishii & Leroy, 2022).

Although the lack of representation of women in leadership roles is often treated as an individual problem (e.g., Sandberg & Scovell, 2014), some argue instead that sex-based bias in organizations is more accurately characterized as a wicked problem (Eden & Wagstaff, 2021), meaning that it is systemic, ambiguous, complex, and conflictual, making appropriate solutions hard to identify and assess (Coe et al., 2019; Eden & Wagstaff, 2021; Madsen, 2021).

In order to see tangible improvements in representation, organizations need to tackle this problem at its core (Leslie, 2019) by ensuring stakeholders—particularly those in hiring positions (often men)—*actually* believe that female leaders are competent in leadership roles (Foley & Williamson, 2019; Furtado et al., 2021; Meyer et al., 2022). Recent surveys suggest that the majority of male MBA students feel that women are under-represented in high-level management roles because they lack the necessary leadership skills (Schenn, 2022). Perceptions of competence are a key criterion in leadership selection, and the subsequent success of individuals in these roles (Chugh & Sahgal, 2007; Cuddy et al., 2011; Dover et al., 2020; Leslie, 2019; Meyer et al., 2022; Tresh et al., 2019; Wentling, 2003). However, little is known about how these perceptions can be influenced for the better, particularly among those with negative sex-based diversity beliefs (i.e., a belief that sex-based diversity is a detriment to the workplace; Chang et al., 2019a, b; Randel, 2023). The limited knowledge in this area is especially problematic given the mounting evidence persistently showing biased evaluations of female leaders’ competence (Atewologun et al., 2018; Begeny et al., 2020; Caleo & Heilman, 2019; Christensen & Muhr, 2019; Dover et al., 2020; Foley & Williamson, 2019; Gangadharan et al., 2016; Ibarra & Obodaru, 2008; Koch et al., 2015; Leslie, 2019; Meyer et al., 2022; Nordell, 2021; Tresh et al., 2019).

Bernstein et al.’s (2020) research focused on how organizations can move from diversity to inclusion to equity, offers an initial step toward addressing this issue. According to their *Theory of Generative Interactions*, organizations may overcome sex-based bias and facilitate inclusion through a set of organizational *practices* that enable prejudice-reducing, adaptive contact among diverse individuals. While leader support is suggested to be essential to this process (Bernstein et al., 2020; Dover et al., 2020; Leslie, 2019; Pettigrew & Tropp, 2006), little guidance is provided on *how* leaders should behave in order to support this change (Nishii & Leroy, 2022; Randel, 2023). Our study suggests diversity-specific empowering leadership (DSEL) may be one viable way forward in attenuating bias in perceptions of female leaders’ competence (particularly among those holding negative sex-based diversity beliefs: Chang et al., 2019a, b; Dover et al., 2020) and in turn, create more inclusive work environments for all.

The literature stresses the importance of having leaders who understand and value diversity in order to successfully manage it within organizations (Byrne et al., 2020; Thomas & Ely, 1996). Additionally, scholars emphasize the need for leaders to avoid backlash if diversity-related changes in the workplace are to succeed (Bernstein et al., 2020; Caleo & Heilman, 2019; Day et al., 2022; Dover et al., 2020; Leslie, 2019). Some research suggests that transformational leadership may be linked to effective diversity management (Ashikali & Groeneveld, 2015; Boerner & Gebert, 2012; Kearney & Gebert, 2009; Mitchell & Boyle, 2009; Ng & Sears, 2012; Okçu, 2014). Other areas of inquiry have found that adapted models of transformational leadership may be particularly effective at targeting change in areas of entrenched behavior (e.g., safety-specific or environmentally specific transformational leadership—Barling et al., 2002; Robertson, 2018). However, the top-down and directive aspects of transformational leadership may also produce backlash and resistance (e.g., Lee et al., 2018; Scheuer & Loughlin, 2021). Further, scholars have issued calls for research to go beyond the entrenched model of transformational leadership, to explore the potential of other models in the context of more collaborative contemporary workplaces (e.g., Guillaume et al., 2017; Lee et al., 2018; Van Knippenberg & Sitkin, 2013).

Most recently, when it comes to generating positive diversity outcomes, empowering leadership has shown promise (Bernstein et al., 2020; Dover et al., 2020; Nishii & Leroy, 2022; Scheuer & Loughlin, 2021). According to Dover et al. (2020), diversity initiatives should be focused more on empowerment than on control, if we are to reduce backlash and resistance. Indeed, Dobbin et al. (2015) found empowering managers (by engaging in diversity promotion and providing autonomy), was the most effective approach in this regard. Consequently, in this study we introduce a target-specific model of leadership adapted to support diversity and inclusion at work—*diversity-specific empowering leadership *(*DSEL*). Further, we compare its effects to alternative models of leadership (general empowering leadership, general and diversity-specific transformational leadership, transactional leadership, leader diversity-valuing behavior, and inclusive leadership). Through their developmental and collaborative approach to diversity-focused to change, diversity-specific empowering leaders (DSELs) may be well positioned to challenge sex-based biases in perceptions of female leaders’ competence (with less backlash), thereby creating more inclusive work environments for everyone (Bernstein et al., 2020; Nishii & Leroy, 2022).

Our study contributes to both theory and practice. First, we advance knowledge on the path from diversity to inclusion by integrating leadership processes into Bernstein et al.’s (2020) Theory of Generative Interactions. Specifically, we demonstrate how DSEL may act as a force that can help overcome exclusionary dynamics (biased perceptions of female leaders’ competence), and in turn foster more inclusive work environments. Recognizing that “change and learning are endemic to inclusion” (Randel, 2023, p. 13), we demonstrate how DSELs collaborative and developmental approach to change encourages *both* men and women to view female leaders as competent through reducing individual resistance and backlash to sex-based diversity (Chang et al., 2019a, b; Day et al., 2022; Dover et al., 2020; Leslie, 2019). Second, our study offers insights into some unintended consequences of diversity initiatives (Caleo & Heilman, 2019; Dover et al., 2020; Leslie, 2019) by introducing sex-based diversity beliefs as a moderator (Randel, 2023) in the link between DSEL and perceptions of female leaders’ competence. Our findings suggest that DSEL may have the greatest impact with individuals whose behavior has been the most resistant to change through existing diversity interventions (i.e., those with negative sex-based diversity beliefs; Atewologun et al., 2018; Chang et al., 2019a, b; Dover et al., 2020; Koch et al., 2015; Meyer et al., 2022; Tresh et al., 2019). Finally, we add to the literature on target-specific leadership by demonstrating the potential of DSEL’s predictive value *above and beyond* general and diversity-specific transformational leadership, along with several other prominent leadership models.

## Literature Review

### Biased Perceptions of Female Leaders’ Competence

Perceptions of female leaders’ competence reflects the extent to which individuals believe women possess the necessary attributes and abilities (e.g., social, emotional, physical, and intellectual/cognitive skills) required to succeed in a role (Kremer et al., 2012). Research suggests that sex-based stereotypes can result in biased perceptions of female leaders’ competence (e.g., Ely et al., 2003; Fiske et al., 2002; Meyer et al., 2022), reducing the chances of women being hired into leadership positions and inhibiting their success in these roles (Cuddy et al., 2011; Dipboye, 1985; Leslie, 2019), ultimately eroding inclusion (Bernstein et al., 2020; Nishii & Leroy, 2022).

Although there is a shift toward more androgynous leadership profiles, or a balance of masculine and feminine traits in leadership (e.g., Powell et al., 2021), leadership is still overwhelmingly associated with masculine/agentic traits (Agut et al., 2022) such as competition, ambition, dominance, independence, assertiveness, and confidence (Eagly, 1987; Eagly & Karau, 2002; Fiske et al., 2002; Heilman, 1983). Indeed, nascent research has examined gendered leadership stereotypes not only in relation to challenges women have in securing leadership roles, but also potentially for gay men (e.g., Liberman & Golom, 2015). While some research finds that gay men may have an advantage in being perceived as high in competence and warmth and therefore equally suitable for either feminine or masculine leadership roles (e.g., Barrantes & Eaton, 2018), other work demonstrates bias towards gay men particularly when rating the suitability for masculine gender-typed work due to gender stereotypes associated with men and specific roles, in addition to prejudice against gay individuals (e.g., Clarke & Arnold, 2018; Pellegrini et al., 2020).

Despite many changes in sex roles over the past 70 years, research shows that gender stereotypes have remained relatively constant with the main difference being that women became viewed as more communal over time whereas men’s agency did not diminish over this time (Eagly et al., 2020). Women’s association with more communal/feminine traits such as being caring, helpful, kind, and cooperative (Eagly, 1987; Eagly & Karau, 2002; Heilman, 1983) creates a disconnect between the leader role (predominately masculine) and female stereotypes (Eagly & Carli, 2007). This role incongruity (Eagly & Karau, 2002) can result in a backlash effect (Rudman, 1998) whereby followers have a negative reaction toward female leaders, including questioning their competence (Meyer et al., 2022; Rudman et al., 2012a, b). As McKinsey & Company (2021a) reported, women must provide more evidence of their competence and have their judgment questioned more than men, even in their areas of expertise (McKinsey & Company, 2021a). This process can result in less access to leadership roles for women (Ferguson, 2018).

Moreover, when an applicant’s credentials are ambiguous, stereotypes are used to fill in the gaps (Darley & Gross, 1983). This allows the criteria which determine a leader’s merit to be defined flexibly (often fitting the perceived strengths of applicants belonging to the favored group), which again can lead to biased perceptions of female leaders’ competence (Uhlmann & Cohen, 2005). Further, these sex-based biases and stereotypes accumulate and can result in large divisions in representation over time. Even small biases can accrue to create the kind of skewed representation we currently see at the top of organizations, with 95% of CEO’s being male (see Nordell, 2021).

Although diversity initiatives are intended to help remedy these problems, research shows that they often backfire by inadvertently increasing rather than decreasing the tendency to behave in a biased manner (Bernstein et al., 2020; Caleo & Heilman, 2019). According to Dover et al. (2020), “the presence of diversity initiatives may signal that underrepresented groups need help to succeed and are thus less competent than their advantaged counterparts” (p. 152). This may contribute to biased perceptions of female leaders' competence and in turn to stalled diversity goal progress. Empirical evidence supports this assertion. For example, early experiments by Heilman et al., (1992, 1997) showed that women and minorities are more likely to be perceived as incompetent when their hiring is presumed to be based on the presence of affirmative action programs. According to attribution theory (Kelley, 1967; Major et al., 1994), the presence of affirmative action policies creates attributional ambiguity about the competence of the groups affected by these policies, making people downplay the impact of their merit or competence (Heilman et al., 1992; Major et al., 1994). Other studies draw similar conclusions, suggesting that diversity initiatives paradoxically may have a negative impact on the perceived competence of women and minorities (Dover et al., 2020; Espino-Pérez et al., 2018; GÜndemir et al., 2017; Heilman & Welle, 2006).

Given the above, identifying alternative approaches that have promise to increase women’s representation in leadership is essential. According to Bernstein et al.’s (2020) Theory of Generative Interactions, organizational practices focused on tackling exclusionary dynamics (e.g., prejudice and stereotyping) and enhancing engagement are key to equality and inclusion in the workplace. However, while leadership is discussed as an essential component in this process (Bernstein et al., 2020; Dover et al., 2020; Leslie, 2019; Pettigrew & Tropp, 2006), it is unclear how leaders should behave to foster these outcomes (Nishii & Leroy, 2022; Randel, 2023). We address this knowledge gap by advancing DSEL as means of overcoming biased perceptions of female leaders’ competence—particularly on the part of those with negative sex-based diversity beliefs (Chang et al., 2019a, b; Dover et al., 2020), and in turn enabling inclusivity.

### The Case for Diversity-Specific Empowering Leadership

**Fig. 1:**
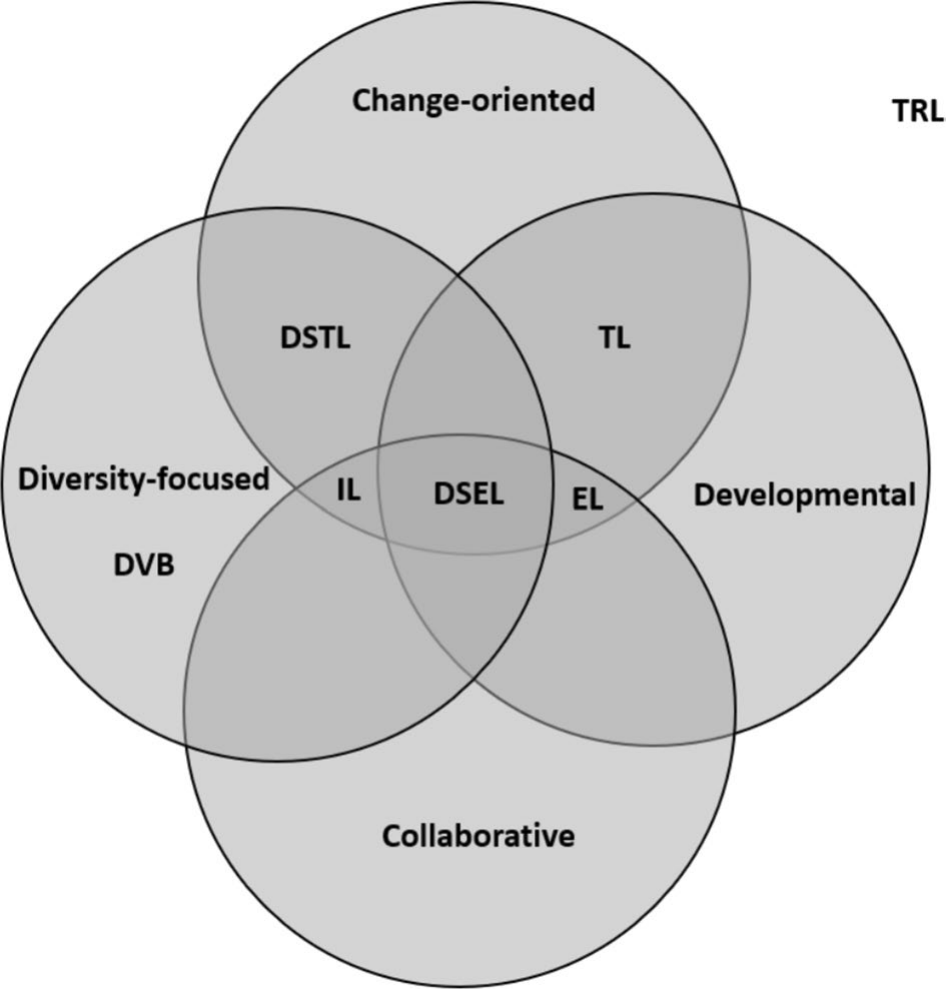
Conceptual similarities and differences of diversity-specific empowering leadership and alternative leadership models. Note: *EL* empowering leadership, *DSEL* diversity-specific empowering leadership, *DVB* Leader diversity-valuing behavior, *TL* transformational leadership, *DSTL* diversity-specific transformational leadership, *IL* inclusive leadership, and *TRL* transactional leadership

A growing body of literature suggests a potential link between sex-based diversity and inclusion at work and various collaborative, inclusive and/or change-focused leadership approaches (e.g., Amabile et al., 2004; Arnold et al., 2000; Carmeli et al., 2010; Hekman et al., 2017; Kearney & Gebert, 2009; Kirkman & Rosen, 1999; Nishii & Leroy, 2022; Scheuer & Loughlin, 2021; Spreitzer et al., 1999; Srivastava et al., 2006; Valerio & Sawyer, 2016; Zhang & Bartol, 2010). In this study, we present a case for DSEL. Although DSEL may share conceptual space with some of these leadership models, an important distinction of DSEL is its ability to circumvent backlash (i.e., defensiveness or resistance) that is often spurred by diversity initiatives (Chang et al., 2019a, b; Day et al., 2022; Dover et al., 2020; Leslie, 2019). This can be accomplished because DSEL blends diversity-focused, change-oriented, collaborative, and developmental behaviors (see Fig. 1). Research suggests that workplace backlash has stronger repercussions toward women leaders compared to men (Rudman & Phelan, 2008). Hence, avoiding backlash is a key factor in achieving sex-based diversity and inclusion (Day et al., 2022; Dover et al., 2020; Leslie, 2019). However, leaders struggle with circumventing this unintended consequence of diversity initiatives.

Research suggests that leaders must go beyond general leadership behaviors if they want to foster sustainable change in this regard (Center for Creative Leadership, 2021). In other words, if a leader leads well in general, and *also* espouses the value of diversity (Dreher, 2003; Vecchio, 2002), this may predict a higher likelihood of employees accepting initiatives aimed at promoting sex-based equity (Day et al., 2022).

This is very similar to approaches used in other areas with entrenched attitudes. For example, Barling et al. (2002) demonstrated the potential of a *safety-specific* transformational leadership model to change intransigent individual attitudes and team climates toward workplace safety. Similarly, Robertson (2018) introduced a model of *environmentally specific* transformational leadership to impact the attitudes and actions of individuals regarding corporate environmental responsibility. This being said, when aiming to overcome backlash associated with diversity-related change (Chang et al., 2019a, b; Day et al., 2022; Dover et al., 2020; Leslie, 2019), recent research suggests that the effects of transformational leadership may be limited.

In Lee et al.’s (2018) meta-analysis of 105 samples, *empowering leadership* was more predictive of outcomes relevant to diversity (such as enhanced creativity, team empowerment, knowledge sharing, organizational citizenship behavior, and trust in leaders), when compared to transformational leadership or leader-member exchange. More recently (again when compared directly to transformational leadership), empowering leadership was a stronger predictor of key diversity outcomes such as information elaboration and team performance (Scheuer & Loughlin, 2021). These results have been attributed to empowering leadership’s focus on collaborative behaviors, as opposed to the directive behaviors associated with transformational leadership (which may elicit backlash). In fact, diversity initiatives concentrated on minimizing managerial discretion can actually have unintended consequences in the form of resistance and backlash (Dover et al., 2020). As a result, scholars (Deci & Ryan, 1987; Dobbin et al., 2015; Dover et al., 2020; Nishii & Leroy, 2022; Pendry et al., 2007) stress the importance of practitioner interventions focused on enhancing autonomy and empowerment and for managers to act in collaborative ways instead of focusing on punitive methods. Other research echoes the conditions of equal status, shared goals, collaboration, and leader support as being powerful in reducing prejudices (Bernstein et al., 2020; Pettigrew & Tropp, 2006). People resist change when their power and control are threatened (Geller, 2003) supporting the need for DSEL. DSELs consider the needs of *all* team members (Nishii, 2013; Nishii & Leroy, 2022; Randel, 2023), thereby avoiding an ‘us vs. them’ mentality which erodes inclusion (Dover et al., 2020).

Similar to the unintended effects of transformational leadership in diverse settings, Hekman et al. (2017) demonstrated that backlash is also associated with ‘diversity-valuing behavior’. Specifically, they showed in both field and laboratory samples, that ethnic minority or female leaders (not white/male leaders) are penalized with worse performance ratings for engaging in explicit diversity-valuing behavior; arguing that this divergent effect results from traditional negative race and sex stereotypes (e.g., lower competence judgments) being placed upon diversity-valuing ethnic minority and female leaders. One unique aspect of DSEL is its explicit attention to how leaders provide followers with autonomy in decision-making (Rafferty & Jimmieson, 2017). This goes beyond respecting diversity among followers (as is done with diversity-valuing behavior; Dwivedi et al., 2023) or highlighting the value of a collective ‘us’ (in the case of transformational leadership). With its collaborative approach to diversity-focused change, DSEL allows followers to openly explore and challenge their views about women’s leadership potential. Importantly, DSELs recognize and foster *everyone’s inclusion* in the change process by actively inviting diverse input and participating in its integration through role modeling (Nishii, 2013; Randel, 2023). Because everyone feels included (even those espousing negative sex-based diversity beliefs), this approach may be more adept at evading the backlash experienced by alternative diversity-focused leadership approaches.

Finally, while inclusive leaders also adopts a collaborative approach to diversity-oriented change, this model is missing an explicit *developmental* component necessary for transformation. Learning and development are a core component of follower openness to change and subsequent adoption of change, and leaders play an essential role in this process (Appelbaum et al., 2015; Geller, 2003; Mousa et al., 2020; Shook et al., 2003). However, while learning and “change seem to be integral components to achieving the kind of transformation involved in inclusion” (Randel, 2023, p. 19), surprisingly, there has been little engagement with this topic in the diversity and inclusion literature. Although some exceptions exist. For example, in the context of age diversity, research has demonstrated how the negative stereotype that older workers are resistant to change may be diminished through leaders educating and supporting older workers through the change process (Scheuer, 2017; Scheuer et al., 2023). According to Randel (2023), “recognition that change and learning are endemic to inclusion as well as being involved in efforts to address the human drive to reduce uncertainty during inclusion-related change may help to inform both theory and practice advancements” (p. 19).

Through the process of empowerment, DSEL’s share their power with others and take steps to develop their followers so that they learn about sex-based biases in non-threatening ways. In supporting followers in their learning and development, DSELs can circumvent the backlash that arises through the frustrations and uncertainty (Berger & Calabrese, 1975; Randel, 2023) associated with learning new skills, particularly when those skills are challenging. Change is unpredictable and comes with complexity which requires organizational members to implement new patterns. For those individuals who hold negative sex-based diversity beliefs this learning and development will be especially challenging, particularly in a context where the representation of women in leadership positions has been historically low. As such, we need a diversity-specific behavioral approach to leadership with a focus on collaboration and active participation (versus directive, dominant or agentic styles) that also support the developmental needs of followers, to ensure leaders proactively prepare their followers for change (Ford & Ford, 2010; Ford et al., 2008; Neves et al., 2021).

Educating employees on how to help reduce unfairness in their workgroup interactions may also elicit less backlash because it permits employees to perceive themselves as change agents rather than targets of bias-reduction programs or policies (Dover et al., 2020). For example, learning how to recognize biased incidents may equip employees to confront them and allow employees to question their behavior without feeling called out (Dover et al., 2020). In this context, DSELs prompt change by encouraging others to lead themselves (Manz & Sims, 1987).

Building upon this literature, we propose that a *diversity-specific model of empowering leadership *(*DSEL*) is a promising lever organizations can use to challenge sex-based biases, specifically regarding female leaders’ competence, without eliciting backlash through a combination of diversity-focused, change-oriented, collaborative, and developmental behavior (see Fig. 1).

### Overcoming Sex-Based Bias and Backlash Through the Components of DSEL

Each of the five components of empowering leadership (Arnold et al., 2000), when focused on diversity, has the potential to attenuate backlash and bias in leadership perceptions. First, based on social learning theory (Bandura, 1986a, b), when DSELs *lead by example*, particularly regarding diversity, the mechanisms related to observational learning (Bandura, 1997) should operate to gently diffuse the leader’s own positive beliefs about female leaders’ competence to their followers (Combs & Luthans, 2007). Through role modeling, DSELs help to reduce ambiguity about, and increase internalization of the inclusive mindsets that are expected of followers (Nishii & Leroy, 2022). Hence, in *leading by example* DSELs help correct biased perceptions and enable inclusion (Bernstein et al., 2020; Nishii & Leroy, 2022). In approaching *change* in this manner, rather than imposing it onto followers, DSELs are also more likely to avoid backlash (Bernstein et al., 2020; Dover et al., 2020). In support of this notion, Day et al. (2022) reported that acting as a positive role model through authentic action was critical for promoting workplace diversity and inclusion and circumventing backlash.

Second, when DSELs *collaborate* through *participative decision-making*, they encourage a more balanced distribution of power, allowing those who have traditionally been excluded from leadership (e.g., womenCoffman, 2014; Konrad et al., 2008; Meyer et al., 2022) to assume both informal and formal leadership roles, and make meaningful contributions that are valued by others. By expressing confidence and supporting voice in those that have traditionally been marginalized (e.g., women), DSELs can help lessen other’s questioning of female leader’s competence (Bernstein et al., 2020; Leslie, 2019; Nishii & Leroy, 2022). With participative decision-making, status characteristics and associated assumptions of incompetence are invalidated, allowing members of historically marginalized groups (e.g., female leaders) to be seen in more complex ways (Nishii & Leroy, 2022, p. 687). Indeed, research demonstrates that providing evidence that targets have high ability (through participative decision-making), lessens bias (Heilman et al., 1997) by preventing the signal that targets need help (Leslie, 2019).

*Participative decision-making* may also help mitigate backlash by inviting input from team members of both sexes when instituting diversity initiatives (Day et al., 2022; Meyer et al., 2022). In encouraging open expression of beliefs and ideals and exploring where these beliefs originated (rather than accusing participants of prejudice), DSELs may alleviate the defensiveness that often arises in some members of the group. By including them in these conversations leaders reinforce the notion that such initiatives are not just creating new biases/injustices (Dover et al., 2020; Leslie, 2019), but rather enabling inclusivity for all.

Third, when DSELs *develop* followers’ *diversity-related* competencies through *coaching,* individuals become more aware of their gender stereotypes and are empowered with the knowledge and skills to counteract their prejudices, thus reducing bias toward non-prototypical (i.e., female) leaders (Day et al., 2022; Meyer et al., 2022). According to Leslie (2019), awareness on the part of ‘majority’ individuals plays a critical role in decreasing the signal that targets (i.e., women) are lacking competence. Other research similarly emphasizes the importance of creating awareness as it may decrease negative reactions to diversity initiatives (e.g., negative spillover; Harrison et al., 2006). Through coaching, DSELs help followers gain the knowledge and skills neccessary to engage in accurate impression formation instead of relying on stereotypical assumptions (Nishii & Leroy, 2022). For example, DSEL can help followers reverse stereotypical beliefs about female leaders’ competence by helping them understand their distinct attributes (i.e., their unique strengths, abilities, and perspectives) and making them visible so that followers can rely on this individuating information rather than superficial assumptions (Nishii & Leroy, 2022; van Dijk et al., 2017). Involving and supporting employees in the learning process of understanding and reducing bias may also result in less backlash as it allows employees to view themselves as catalysts for change (Appelbaum et al., 2015; Dover et al., 2020).

Crisp and Turner’s ‘Categorization-Processing–Adaptation–Generalization Model’ (2010) highlights the importance of not only knowledge and awareness, but also motivation, repetition, and adaptive learning in shifting stereotypical inconsistencies. Studies in the diversity and inclusion and change literatures support this notion (Gilley et al., 2009; Nishii & Leroy, 2022; Shook et al., 2003). For example, Appelbaum et al. (2015) argued that, “leadership skills and abilities are positively associated with success in executing change—being capable of communicating, coaching, involving others, all in order to motivate, reward and build teams, and calls for the critical ability to recognize and respond to individual needs during change” (p. 138). Through coaching, DSELs can motivate individuals to become more aware of their biases, allowing them to adapt their attitudes and behaviors with repetition/practice, and in turn, to reduce prejudice (Bernstein et al., 2020).

Fourth, DSELs may also support followers through the *change* process by *showing concern* for those who have been marginalized (e.g., women in this case) and for those who perceive they *could be* marginalized (e.g., white men). Showing concern for minority group members (e.g., female leaders) can help reduce anxieties and self-doubts about their competence and produce greater comfort in interactions (Bernstein et al., 2020; Dover et al., 2020). Further, empathizing with the majority group (in this case men) is also important because men can feel victimized by these initiatives. As mentioned, the majority of MBA men actually perceive that *they* will be the victims of bias, despite the low number of women holding senior leadership roles (Schenn, 2022). Further, Dover et al. (2020) argue that the mere presence of diversity programs or policies can lead majority group members to perceive that the organization prioritizes minorities over non-minorities and can cause non-minorities to attribute minorities' career advancement to ‘reverse discrimination’ (p. 163). By DSELs alleviating these concerns, backlash associated with diversity initiatives could be avoided (Dover et al., 2020).

Finally, DSELs can enable *diversity-focused change* by ensuring their followers are properly *informed* of goals, policies, and procedures around the importance of diversity (Dover et al., 2020). Randel (2023) similarly highlighted the importance of leaders sharing information to increase diversity initiative effectiveness. By informing others of both the moral and business case for diversity, DSELs can help reduce the likelihood of backlash directed towards non-traditional (i.e., female) leaders, in turn, improving recognition of their competence (Dover et al., 2020). Consequently, we hypothesize:

#### H1:

DSEL will be positively related to perceptions of female leaders’ competence.

### Sex-Based Diversity Beliefs as a Moderator

Research calls for an exploration of the moderating effects of diversity beliefs as a means of understanding the path to equity and inclusion (Randel, 2023). As discussed earlier, biases against female leaders persist (Atewologun et al., 2018; Gangadharan et al., 2016; Ibarra & Obodaru, 2008; Koch et al., 2015; Tresh et al., 2019), particularly among those with negative sex-based diversity beliefs (Van Dick et al., 2008). Hence, we incorporate sex-based diversity beliefs as a moderator to help explain when and with whom, DSELs may prompt change in perceptions of female leaders’ competence. Sex-based diversity beliefs reflect the extent to which followers’ value and see the benefits of sex-based diversity at work (Meyer et al., 2022). Whereas perceptions of female leaders’ competence involve prejudice surrounding the perceived capabilities of a specific group (i.e., female leaders), sex-based diversity beliefs capture broader attitudes toward sex-based diversity. Individuals with *positive sex-based diversity beliefs* view sex-based diversity as a benefit to team functioning and success. These individuals appreciate the contributions of each individual team member, and so are less likely to favor one sex over the other based on biased perceptions of competence (Meyer et al., 2022). In contrast, individuals with *negative sex-based diversity beliefs* do not see the benefits of sex-based diversity at work, instead viewing it as a hindrance.

Individuals with negative sex-based diversity beliefs should be more likely to hold negative perceptions of female leaders’ competence and in some cases also perceive that female leaders (i.e., the minority sex in terms of traditional leadership roles) pose a threat to their career progress triggering notions of reverse discrimination (Dover et al., 2020). This group of individuals may be more likely to respond to diversity efforts with backlash (Chang et al., 2019a, b; Dover et al., 2020; Meyer et al., 2022). However, given these individuals currently hold negative beliefs about sex-based diversity, there may also be a unique opportunity for DSELs to have a greater impact on these individual’s biased perceptions of female leaders’ competence than for those who already believe sex-based diversity will be positive for the workplace.

Some research shows that attempts to reduce prejudice will only be successful for those already accepting of diversity (Dover et al., 2020). Through their collaborative and development approach to diversity-focused change, DSELs can circumvent much of the defensiveness that often arises from individuals with negative sex-based diversity beliefs (Chang et al., 2019a, b). Since individuals with negative sex-based diversity beliefs have a steeper learning curve and experience greater uncertainty (Berger & Calabrese, 1975) with respect to correcting biased perceptions of female leaders’ competence they are more in need of engagement and support during the change process, making the effects of DSEL more pronounced for this group of individuals. A similar relationship has been observed in the context of age diversity and the adoption of new technologies. Although both younger and older workers may benefit from having an empowering leader in these circumstances, older workers responded even more strongly to these behaviors due the adoption of new technologies posing a greater challenge for this age group (Scheuer, 2017; Scheuer et al., 2023). Hence, through DSEL’s collaborative and developmental approach to diversity-focused change, these types of leaders may enable a cognitive shift (Thomas et al., 1990) in attitudes towards female leaders especially among those most resistant to it (i.e., individuals with negative sex-based diversity beliefs). Consequently, we hypothesize:

#### H2:

Sex-based diversity beliefs will moderate the positive relationship between DSEL and perceptions of female leaders’ competence, such that the relationship will be stronger for those individuals with negative sex-based diversity beliefs.

### Relationship of DSEL and Perceptions of Female Leaders’ Competence with Climate for Inclusion

Climate for inclusion was initially defined by Nishii (2013) as employee perceptions of the extent to which organizational policies and practices promote and reward inclusive behaviors focused on fostering uniqueness and belongingness of employees from diverse demographic backgrounds (Mor Barak, 2005; Mor Barak et al., 2022; Nishii, 2013; Schneider, 1990; Shore et al., 2011). Later, Nishii and Leroy (2022) updated this description to include the needs for competence (i.e., felt mastery over tasks that help bring about desired outcomes) together with belonging (i.e., a sense of relational security) and autonomy (i.e., internal locus of control over the expression of one’s thoughts and behaviors), in order to experience inclusion at work.

An extensive meta-analysis (Barak et al., 2016) from the last 20 years of research on diversity and inclusion found that managerial efforts to enhance perceptions of *inclusion* were consistently related to positive outcomes. Additionally, corporate governance research (Guldiken et al., 2019) has established a positive association between inclusive climates and sex-based equity in leadership. Hence, in order for organizations to realize meaningful representation of women in leadership, we should likely focus our efforts on proximal drivers (i.e., antecedents and mediators) of inclusive work environments (Randel, 2023). Ample evidence suggests an association between mitigating interpersonal bias and creating a sustainable climate for inclusion (Caleo & Heilman, 2019; Nishii, 2013).

In this study, we focus on biases associated with female leaders’ competence as an explanation for how DSEL influences an inclusive climate. In their Theory of Generative Interactions, Bernstein et al. (2020) posit that exclusionary dynamics (e.g., sex-based bias) will predominate by default if not countered by other forces. These forces include: (1) Shared Organizational Purpose, (2) Intentional Community Building, (3) Frequent Interactions, (4) Equal Status in Decision-Making, (5) Collaboration with Member Interdependence while Valuing Unique Needs, and (6) Interpersonal Comfort & Self-Efficacy. Based on the arguments presented earlier, we theorize that DSELs create these necessary conditions for overcoming bias and enabling inclusion.

For example, at the heart of DSEL is collaboration and power sharing [linked to Bernstein et al.’s (2020) conditions 2–5 listed above]. Shore et al. (2011) confirm that for inclusion to be fostered, we need to support practices associated with promoting ‘insider status’, including sharing information, participation in decision-making, and supporting voice. Interpersonal comfort and self-efficacy may be realized through DSELs coaching and showing concern for the whole team. As Bernstein et al. (2020) noted, “organizational practices [such as coaching] that help individuals become more skilled in cross-cultural interactions should aid in reducing anxieties and distancing, producing greater comfort in interactions” (p. 399). Through showing concern, DSELs can also support the efficacy of their followers by challenging “existing assumptions about the relationship between social identities and competence, and in so doing delegitimize arbitrary status hierarchies and improve followers’ experiences of inclusion vis-`a-vis the leader as well as workgroup members” (Nishii & Leroy, 2022, p. 692). Finally, a shared organizational purpose can be enabled through the informing component of DSEL. As Nishii and Leroy (2022) argued, in order to enable inclusion leaders at every level must inform followers of diversity-related goals, policies, and procedures. Consequently, drawing on Bernstein et al.’s (2020) framework and the other arguments presented above, we propose that DSEL may serve as a force that can help overcome exclusionary dynamics (i.e., biased perceptions of female leaders’ competence), and in turn foster more inclusive work environments.

While the above evidence supports an indirect relationship between DSEL and a climate for inclusion (via perceptions of female leaders’ competence), research suggests DSEL may also directly impact a climate for inclusion. Followers tend to understand the behavioral expectations in the workplace based on the day-to-day interactions with their leaders (Schneider et al., 1998). Boekhorst (2015) argues that leaders who convey social information on the importance of including employees in work processes (which occurs through DSEL’s collaborative behaviors) can directly enhance an environment of inclusion. Recent work by Shore and Chung (2023) suggests that leaders who lead by example by exhibiting inclusive behaviors and facilitating social interactions among followers (both aspects of DSEL), mitigate social exclusion and enhance perceptions of workgroup inclusion. As Nishii and Leroy (2022) argued, inclusion and engagement result when individuals are *empowered* to contribute, knowing that their co-workers will notice their competence and understand where they are coming from (e.g., value their social identities and previous experiences). In this way “… needs for autonomy, belonging, and competence are satisfied.” (p. 688). Moreover, followers tend to perceive empowering leaders as credible because these leaders support followers' basic needs for autonomy and competence and create effective working conditions for engagement (e.g., Tuckey et al., 2012).

Hence, we propose:

#### H3:

DSEL will be both indirectly (via perceptions of female leaders’ competence) and directly positively associated with a climate for inclusion.

### Mediated Moderation Model

In summary, grounded in Bernstein et al.’s (2020) Theory of Generative Interactions and drawing upon the other arguments and research presented above, we propose a mediated moderation model whereby the relationship between DSEL (predictor) and perceptions of female leaders’ competence (mediator) is moderated by sex-based diversity beliefs which, in turn, are associated with climate for inclusion. This mediated moderation model demonstrates how DSEL can play a transformative role in shaping inclusive organizational climates by addressing individual biases and reinforcing inclusive norms, especially for those holding negative sex-based diversity beliefs.

#### H4:

Sex-based diversity beliefs will moderate the indirect effect of DSEL on climate for inclusion, via perceptions of female leaders’ competence; among those with negative sex-based diversity beliefs the effect will be stronger.

**Fig. 2:**
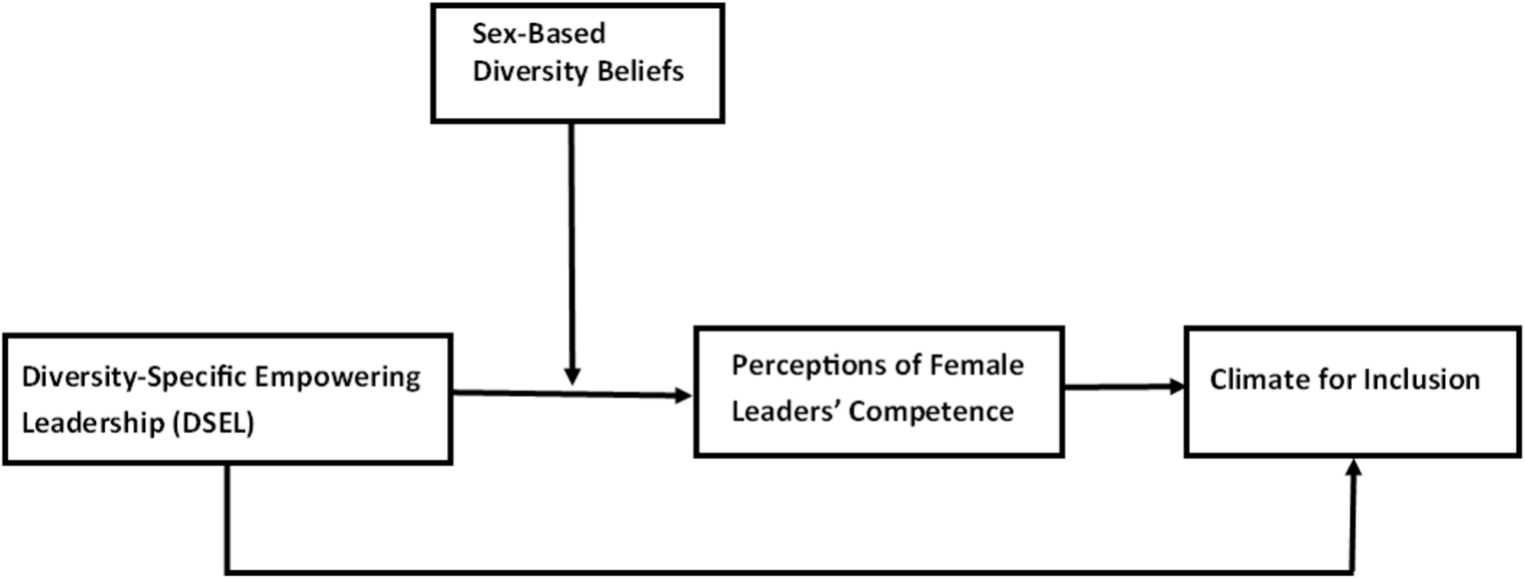
Research model

Figure 2 illustrates the proposed relationships among the main variables in our study.

## Methods

### Overview

We conducted three studies to test our hypotheses. In Study 1 (*N* = 236), we develop and test a model of DSEL and compare this new measure to alternative leadership models (empowering leadership, transformational leadership, diversity-specific transformational leadership, transactional leadership, leader diversity-valuing behavior, and inclusive leadership) in its ability to predict perceptions of female leaders’ competence including using two supplemental samples (*N* = 313 and *N* = 510). In Study 2, using a fourth sample (*N* = 314), we replicate Study 1 and explore a potential moderator (i.e., sex-based diversity beliefs) in the relationship between DSEL and perceptions of female leaders’ competence. Finally, in Study 3 we draw upon one of the supplemental samples from Study 1 (*N* = 313), to test a mediated moderation model with sex-based diversity beliefs moderating the indirect effect of DSEL on climate for inclusion (via perceptions of female leaders’ competence). Specifically, Study 1 tests Hypothesis 1. Study 2 replicates Hypothesis 1 and tests Hypothesis 2. Finally, Study 3 replicates hypotheses 1 and 2 and tests hypotheses 3 and 4.

## Study 1

### Sample and Procedure

Participants were recruited using Qualtrics, an approach that has been used successfully in prior management research (e.g., Triana et al., 2017; Walter et al., 2019). Individuals were full-time employees and had held their current positions, reporting to the same supervisor for a minimum of 1 year. Participants were incentivized with redeemable points for prizes. Participants were employed in an organization based in the U.S. or Canada, and worked in a variety of industries (mean = 3.7 years of experience in their current organizations). Participant ages ranged from 18 to 73 years (mean = 52 years); 50% of employees were female, and 44.3% of the raters reported to female leaders.

#### Measures

Empowering Leadership (*α* = 0.96) was measured using the 15-item Empowering Leadership Questionnaire (ELQ; Arnold et al., 2000; Srivastava et al., 2006). Participants rated their leaders on items such as: “Leads by example” and “Supports the efforts of our team.” Each item was rated on a 5-point scale (1 = Not at all, 5 = Frequently if not always).

Diversity-Specific Empowering Leadership (DSEL) was measured using a 15-item adapted version of the ELQ (Arnold et al., 2000; Srivastava et al., 2006). The scale showed internal reliability of *α* = 0.97. Following Barling et al.’s (2002) strategy, each of the items was modified to address the diversity-specific context of the study (e.g., “Leads by example” was changed to “Leads by example when it comes to diversity and inclusion” and “Supports the efforts of our team” was changed to “Supports our team’s diversity and inclusion efforts”). Each item was rated on a 5-point scale (1 = Not at all, 5 = Frequently if not always). We established face validity through the analysis of interviews with leaders and their followers, and demonstrated the reliability and factor structure (i.e., construct validity) using confirmatory methods for the new measure. Further details of the DSEL scale development process and a listing of the items can be found in Appendices A and B, respectively.

Perceptions of Female Leaders’ Competence (*α* = 0.90) was measured using a five-item scale developed by Hekman et al. (2017). Sample items include: “To what extent do you think of female leaders as: Capable—competent and qualified for top-level positions; and Effective—in getting projects done well and on time”. Responses were provided on a five-point scale (1 = Never, 5 = Always).

Positive Prior Exposure to Female Leaders was included as a control, due to evidence suggesting prior positive exposure to women in leadership positions may help mitigate bias against female leaders (Afridi et al., 2013; Allport, 1954; Beaman et al., 2009, 2012; Bohnet, 2016; Chalise et al., 2021; Chetty et al., 2016; Gangadharan et al., 2016; Lawson et al., 2022; Nordell, 2021; Pettigrew et al., 2011). For example, in their study of 61 villages in India, Gangadharan et al. (2016) found that male bias towards female leaders tended to disappear with greater exposure. Lawson et al. (2022) found that hiring women into leadership positions mitigated deep-rooted stereotypes that are expressed in language, which led to women being associated with characteristics that are critical for leadership success, including competence. This construct was measured based on Bohnet (2016): “My prior experience working with female leaders has largely been positive”. It was rated on a 5-point scale (1 = Disagree, 5 = Agree).

Rater and Leader Sex were also included as controls since prior research has demonstrated that demographic variables (Randel, 2023) such as rater (Atewologun et al., 2018; Koch et al., 2015; Tresh et al., 2019) and leader sex (Eagly & Karau, 2002; Ferguson, 2018; Johnson et al., 2008; McKinsey & Company, 2021a; Rudman & Glick, 2001) matter when it comes to understanding the effects of leadership behavior on diversity outcomes. For example, male raters have been found to rate female leaders less favorably than they rate male leaders (Atewologun et al., 2018; Koch et al., 2015; Tresh et al., 2019). Moreover, male leaders are more likely than female leaders to have their leadership endorsed (Eagly & Karau, 2002; Ferguson, 2018; Gangadharan et al., 2016; Ibarra & Obodaru, 2008; Johnson et al., 2008; McKinsey & Company, 2021a; Rudman & Glick, 2001) and are less prone to backlash when engaging in diversity-valuing behavior (Hekman et al., 2017). Notably, in McKinsey & Company’s (2021a) “Women in the Workplace 2021” study, women were more likely to have their competence questioned and their authority undermined. See Appendix C for details on preliminary analyses conducted to justify inclusion of these controls.

### Results

#### Predictive Validity: Hypothesis Testing

Table 1 presents the means, standard deviations, and bivariate correlations for all variables used in hypothesis testing.

**Table 1:** Overall bivariate correlations between main variables in Study 1

Scale	*M*	SD	1	2	3	4	5	6
1. Rater sex	0.5	0.50		0.56**	0.05	0.20**	0.08	0.02
2. Leader sex	0.56	0.50			0.05	0.02	0.04	0.11
3. Empowering leadership	3.91	0.91				0.23**	0.81**	0.37**
4. Positive prior exposure to female leaders	4.42	0.99					0.23**	0.46**
5. Diversity-specific empowering leadership	3.70	1.03						0.40**
6. Perceptions of female leaders’ competence	4.12	0.66						

*Correlation is significant at the 0.05 level (2-tailed)

**Correlation is significant at the 0.01 level (2-tailed). *N* = 236

To test whether DSEL was positively related to perceptions of female leaders’ competence, we carried out a standard regression analysis using SPSS Version 27. We entered rater sex, leader sex, positive prior exposure to female leaders, empowering leadership (EL) and DSEL into the regression model predicting perceptions of female leaders’ competence. Analysis showed statistical significance, *F*(5, 224) = 24.856, *p* < 0.001, and this accounted for 36% of the predicted variance. Specifically, rater sex had a significant and negative relationship with perceptions of female leaders’ competence (*β* = − 0.240, *p* = 0.006), meaning male raters had more negative perceptions of female leaders. In contrast, leader sex (being male; *β* = 0.256, *p* = 0.003), having positive prior exposure to female leaders (*β* = 0.265, *p* < 0.001) and DSEL (*β* = 0.125, *p* = 0.031) were positively related to perceptions of female leaders’ competence. The effects of EL was not significant (*β* = 0.116, *p* = n.s.). DSEL accounted for an additional 10.3% (*p* < 0.001) of the explained variance above and beyond the effects of the control variables. Thus, Hypothesis 1 was supported.

### Supplemental Analysis

Supplemental analyses were performed using additional samples which showed that the effects of DSEL were distinct from other similar leadership models, namely transactional leadership, empowering leadership, transformational leadership, diversity-valuing behavior, inclusive leadership, and a newly developed diversity-specific transformational leadership (DSTL) measure. See Appendix C for details.

## Study 2

### Method

Data (*N* = 314) were collected using the same criteria and procedures outlined in Study 1. Participants resided in either the U.S. or Canada and worked in a variety of industries (mean = 3.3 years of experience in their respective organizations). Participants’ ages ranged from 19 to 69 years (mean age = 41 years; 48.7% female.). Of the raters, 42.7% reported to female leaders.

#### Measures

We used the same measures as Study 1 for empowering leadership (*α* = 0.96), DSEL (*α* = 0.97), perceptions of female leaders’ competence (*α* = 0.90), rater sex, leader’s sex, and positive prior exposure to female leaders.

Sex-Based Diversity Beliefs (*α* = 0.73) were also assessed using a four-item 5-point scale (1 = Strongly disagree, 5 = Strongly agree; Van Dick et al., 2008). Items included: “I think that teams benefit from the involvement of a mix of both men and women” and “Creating teams that contain a mix of both men and women can be a recipe for trouble”. Negatively worded items were recoded and mean scores created. Low scores on this measure (below three) indicate negative sex-based diversity beliefs while high scores (above three) indicate positive sex-based diversity beliefs.

### Results

#### Descriptive Statistics and Correlations

Table 2 presents the means, standard deviations, and bivariate correlations for all variables used in hypothesis testing.

**Table 2:** Overall bivariate correlations between variables in Study 2

Scale	*M*	SD	1	2	3	4	5	6	7
1. Rater sex	0.5	0.50		0.44**	0.06	0.18**	0.03	− 0.08	− 0.23**
2. Leader sex	0.57	0.50			0.03	− 0.01	0.03	− 0.03	− 0.18**
3. Empowering leadership	3.93	0.91				0.17**	0.83**	0.31**	0.19**
4. Positive prior exposure to female leaders	4.37	1.04					0.21**	0.47**	− 0.02
5. Diversity-specific empowering leadership	3.78	1.00						0.37**	0.15**
6. Perceptions of female leaders’ competence	4.14	0.65							0.20**
7. Sex-based diversity beliefs	3.75	0.98							

*Correlation is significant at the 0.05 level (2-tailed)

**Correlation is significant at the 0.01 level (2-tailed). *N* = 314

#### Hypothesis 1 Replication Testing

To re-test the relationship between DSEL and perceptions of female leaders’ competence, we regressed the control variables (rater sex, leader’s sex, positive prior exposure to female leaders, EL, and DSEL) and predictor variables (DSEL) onto the outcome variable (perceptions of female leaders’ competence). Analysis showed statistical significance, *F*(5, 303) = 28.99, *p* < 0.001, and this accounted for 32.4% of the predicted variance. Rater’s sex once again showed a negative relationship with perceptions of female leaders’ competence (*β* = − 0.20, *p* < 0.001), whereas leader’s sex (*β* = 0.047, *p* = n.s.) and empowering leadership (*β* = 0.02, *p* = n.s.) were non-significant. Further, positive prior exposure to female leaders (*β* = 0.447, *p* < 0.001) and DSEL (*β* = 0.253, *p* = 0.003) were positively related to perceptions of female leaders’ competence. DSEL accounted for an additional 5% (*p* < 0.001) of the explained variance above and beyond the effects of the control variables. Thus, Hypothesis 1 was again supported.

#### Moderation Effects of Sex-Based Diversity Beliefs (Hypothesis 2)

**Fig. 3:**
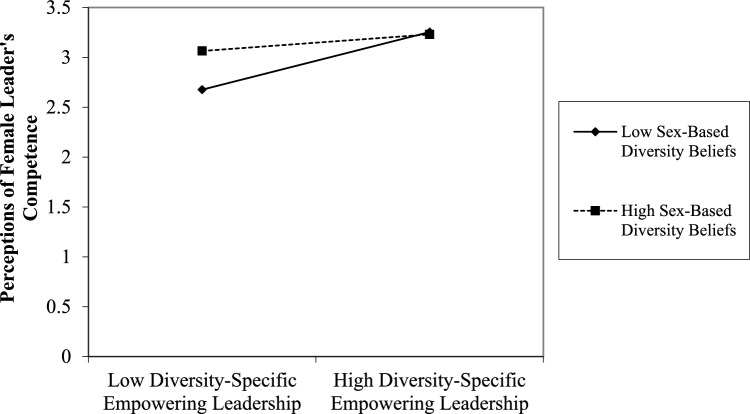
Plot of interaction of diversity-specific empowering leadership and sex-based diversity beliefs predicting perceptions of female leader’s competence in Study 2

To test Hypothesis 2, we conducted moderated regression analysis as outlined by Stride (2017). Using Mplus Version 8, we regressed the dependent variable (perceptions of female leaders’ competence) on the independent variable (DSEL), the moderator (sex-based diversity beliefs) and the interaction between the independent and the moderator variables, controlling for rater and leader sex and prior positive exposure to female leaders. All exogenous variables were mean centered prior to conducting our analysis (Stride, 2017). Additionally, to interpret the effect of our interaction term, as per Stride’s (2017) recommendation, the value of the moderator variable (sex-based diversity beliefs) was entered at one standard deviation above and below its mean. In our case, low levels of sex-based diversity beliefs equated to a value of 2.77, which falls on the disagreement side of the sex-based diversity beliefs scale, representing those with negative sex-based diversity beliefs. While high levels of sex-based diversity beliefs equated to a value of 4.73, which falls on the agreement side of the sex-based diversity beliefs scale, representing those with positive sex-based diversity beliefs. Sex-based diversity beliefs moderated the relationship between DSEL and perceptions of female leaders’ competence (*b* = − 0.103, *p* = 0.016, *R*^2^ = 0.36). The moderation effects also explained variance above and beyond what was attributed to the main effects of the predictor variables (Δ*R*^2^ = 0.02, *p* = 0.003). The statistical significance of the interaction term and associated effect sizes underscores the meaningful role of sex-based diversity beliefs in moderating DSEL’s impact. It demonstrates that this moderator contributes unique explanatory power in predicting perceptions of female leaders’ competence. Additionally, simple slope analysis methodologically strengthens our findings by showing that DSEL’s impact on perceptions of female leaders’ competence varies systematically with individual diversity beliefs. Specifically our results showed that, at low levels of sex-based diversity beliefs (i.e., for individuals perceiving sex-based diversity to be a detriment in the workplace), DSEL was more strongly associated with perceptions of female leaders’ competence (*b* = 0.289*, p* < 0.001) than for those with high levels of sex-based diversity beliefs, i.e., for individuals that perceive sex-based diversity to be beneficial to the workplace, (*b* = 0.083*, p* = 0.08), thereby supporting Hypothesis 2. See Fig. 3.

### Supplemental Analysis

We conducted two supplemental moderation analyses, one with rater sex as a moderator (when controlling for leader sex) and the other with leader sex as a moderator (when controlling for rater sex) in the relationship between DSEL and perceptions of female leaders’ competence. Findings suggested DSEL had stronger effects on perceptions of female leaders’ competence for male raters and male leaders. See Appendix D for details.

## Study 3

### Method

The sample for Study 3 was taken from Study 1’s datasets (Supplemental Analysis, fourth sample). Data (*N* = 313) were collected using the same criteria and procedures outlined in Studies 1 and 2. Participants resided in either the U.S. or Canada and worked in a variety of industries. Participants’ ages ranged from 18 to 59 years; 51.4% female. Of the raters, 44.4% reported to female leaders.

### Measures

We used the same measures as before for DSEL (*α* = 0.97), perceptions of female leaders’ competence (*α* = 0.93), rater sex, and leader’s sex.

Climate for Inclusion (*α* = 0.96) was assessed using the 15-item measure developed by Nishii (2013). An example item was “This organization has a culture in which employees appreciate the differences that people bring to the workplace”. Participants rated the items on a 5-point scale (1 = Strongly disagree, 5 = Strongly agree).

Prior exposure to female leaders was measured using a slightly modified version of the *positive* prior exposure measure used in studies 1 and 2. This was done to account for the possibility of individuals having prior exposure with female leaders that was not necessarily positive, as studies have shown that negative interactions in groups can also have important effects on diversity outcomes (Graf & Paolini, 2016). The measure was based on Bohnet (2016) with the stem and scales reading: “My prior experience working with female leaders (excluding your current manager/supervisor) has been”: (1 = largely negative to 5 = largely positive).

### Results

#### Descriptive Statistics and Correlations

Table 3 presents the means, standard deviations, and bivariate correlations for all variables used in hypothesis testing.

**Table 3:** Overall bivariate correlations between variables in Study 3

Scale	*M*	*SD*	1	2	3	4	5	6	7
1. Rater sex	0.51	0.50		0.37**	− 0.20**	0.00	− 0.02	− 0.10	0.10
2. Leader sex	0.44	0.50			0.00	0.01	0.06	− 0.17**	0.11
3. Prior exposure to female leaders	3.74	1.23				0.32**	0.55**	0.28**	− 0.00
4. Diversity-specific empowering leadership	3.69	1.02					0.42**	0.71**	0.01
5. Perceptions of female leaders’ competence	3.89	0.68						0.39**	0.16**
6. Climate for Inclusion	3.70	0.85							− 0.10
7. Sex-based diversity beliefs	3.72	0.90							

*Correlation is significant at the 0.05 level (2-tailed)

**Correlation is significant at the 0.01 level (2-tailed). *N* = 313

### Hypothesis 1 Replication Testing

To confirm the relationship between DSEL and perceptions of female leaders’ competence, we regressed the control variables (rater sex, leader’s sex, prior exposure to female leaders, and predictor variable (DSEL) on the outcome (perceptions of female leaders’ competence). Analysis showed statistical significance, *F*(4, 289) = 42.90, *p* < 0.001, and this accounted for 37% of the predicted variance. DSEL (*β* = 0.291,* p* < 0.001) and prior exposure to female leaders (*β* = 0.476, *p* < 0.001) were positively related to perceptions of female leaders’ competence offering additional support for our first hypothesis. DSEL explained an additional 6% of the variance (*p* < 0.001) beyond what was captured by the other variables in the model.

### Effects of DSEL on Perceptions of Female Leaders’ Competence and Climate for Inclusion

To test whether perceptions of female leaders’ competence partially mediates the relationship between DSEL and climate for inclusion (Hypothesis 3), we conducted mediation analysis as outlined by Kelloway (2014) and MacKinnon et al. (2007). Using Mplus Version 8, we regressed the dependent variable (climate for inclusion) on the predictor variable (DSEL), and the mediator variable (perceptions of female leaders’ competence) on the predictor variable (DSEL), while controlling for leader/rater sex and prior experience with female leaders. We calculated the product of the paths from the predictor (DSEL) to the mediator variables (perceptions of female leaders’ competence), and the path from the mediator (perceptions of female leaders’ competence) to the dependent variable (climate for inclusion). To test the statistical significance of the indirect effect, we calculated 95% credibility intervals using the Bayes estimator in Mplus (Muthén & Muthén, 2014). The indirect (mediated) effect of DSEL on climate for inclusion via perceptions of female leaders’ competence was significant, *b* = 0.04, SE = 0.02, 95% CI (0.004, 0.08), *R*^2^ = 0.52. Since the significant direct effect of DSEL on climate for inclusion was still significant after controlling for the mediator (*b* = 0.540*, p* < 0.001), these results demonstrate partial rather than full mediation. Thus, Hypothesis 3 was supported.

### Mediated Moderation Model (Hypothesis 2 and 4)

**Fig. 4:**
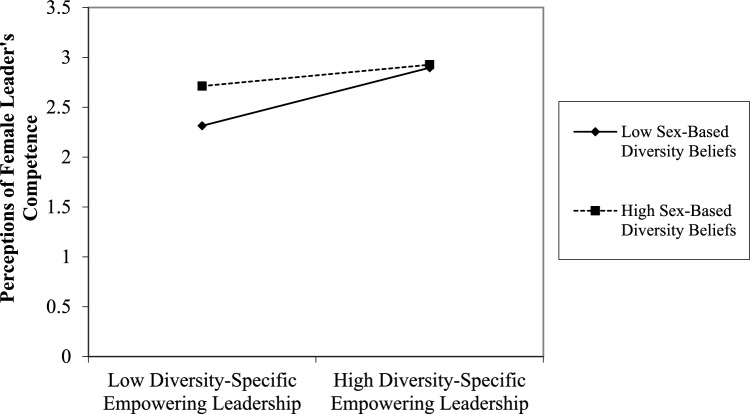
Plot of interaction of diversity-specific empowering leadership and sex-based diversity beliefs predicting perceptions of female leader’s competence in Study 3

Hypothesis 4 posits a mediated moderation effect, which occurs when the interaction between two variables affects a mediator, which in turn is associated with a dependent variable. To test Hypothesis 4, we followed procedures outlined by Morgan-Lopez and MacKinnon (2006) and Kearney and Gebert (2009). We regressed the mediator (perceptions of female leaders’ competence) on the controls (participant/leader sex and prior experience with female leaders), the independent (DSEL) and moderator variable (sex-based diversity beliefs), and the interaction between the independent variable (DSEL) and the moderator (sex-based diversity beliefs). We regressed the dependent variable (climate for inclusion) on the controls, mediator, independent and moderator variable, and the interaction between the independent variable and the moderator. All exogenous variables were mean centered prior to computing interaction terms (Aiken & West, 1991). Additionally, following Stride’s (2017) recommendation, to interpret the effect of our interaction term the value of the moderator variable (sex-based diversity beliefs) was entered at one standard deviation above and below its mean. In our case low levels of sex-based diversity beliefs equated to a value of 2.82, which falls on the disagreement side of the sex-based diversity beliefs scale, representing those with negative sex-based diversity beliefs. While high levels of sex-based diversity beliefs equated to a value of 4.62, which falls on the agreement side of the sex-based diversity beliefs scale, representing those with positive sex-based diversity beliefs. To estimate the indirect (mediated moderation) effects, we calculated the product of the path from the interaction term to the mediator and the path from the mediator to the dependent variable (Kearney & Gebert, 2009; Morgan-Lopez & MacKinnon, 2006). To test the statistical significance of the respective indirect effects, we calculated 95% credibility intervals using the Bayes estimator in Mplus (Muthén & Muthén, 2014). The results demonstrated significant moderating effects of sex-based diversity beliefs on the relationship between DSEL and perceptions of female leaders’ competence (*b* = − 0.092, *p* = 0.028, *R*^2^ = 0.401, Δ*R*^2^ = 0.01, *p* = 0.02). Additionally, simple slope analysis showed that, at low levels of sex-based diversity beliefs (i.e., for individuals perceiving sex-based diversity to be a detriment to the workplace), DSEL was more strongly associated with perceptions of female leaders’ competence (*b* = 0.282*, p* < 0.001) when compared to those with high levels of sex-based diversity beliefs, (*b* = 0.116*, p* = 0.015), thereby offering additional support for Hypothesis 2. See Fig. 4. The indirect (mediated) effects of the interaction of DSEL with sex-based diversity beliefs via perceptions of female leaders’ competence on climate for inclusion was also significant, *b* = − 0.018, SE = 0.01, 95% CI (− 0.041, − 0.002), *R*^2^ = 0.40 (perceptions of female leaders’ competence), *R*^2^ = 0.55 (climate for inclusion)*.* Thus, Hypothesis 4 was also supported.

### Supplemental Analysis

In recognizing that eliminating bias and discrimination is considered to be a precondition for inclusion experiences (Nishii & Leroy, 2022), we treated perceptions of female leaders’ competence as a mediator in the relationship between DSEL and a climate for inclusion. That said, while the constructs in our paper have been treated in this manner (since this is how they have been conceptualized in the literature; Randel, 2023), alternative pathways are certainly still plausible; for instance, a favorable climate for inclusion (in conjunction with a DSEL) may attenuate biased perceptions of female leaders’ competence (Caleo & Heilman, 2019; Nishii, 2013). The results did not support such an alternative model when considering the moderating effects of sex-based diversity beliefs. See Appendix E for details.

## Discussion

Our study offers insight into the inclusion policy/performance gap in organizations (Eden & Wagstaff, 2021; The Conference Board of Canada, 2013; Madsen, 2021; Nishii & Leroy, 2022) by examining how DSEL might overcome biased perceptions of female leaders’ competence (particularly for those with negative sex-based diversity beliefs) and foster a more inclusive work enviornment. In Study 1, we developed and validated a new DSEL measure and demonstrated its value for predicting perceptions of female leaders’ competence when compared to alternative models of leadership (empowering leadership, transformational leadership, DSTL, transactional leadership, leader diversity-valuing behavior, and inclusive leadership) using two additional samples (*N* = 313 and *N* = 510). The incremental contributions of DSEL when compared to these other leadership models could be explained by its collaborative and developmental approach to diversity-oriented change which serves to encourage *and* support *both* men and women’s engagement in the change process thereby reducing the tendency for backlash against such efforts (Chang et al., 2019a, b; Day et al., 2022; Dover et al., 2020; Leslie, 2019; Nishii & Leroy, 2022).

In Study 2, we demonstrated that the positive effects of DSEL on perceptions of female leaders’ competence were more pronounced for individuals with negative sex-based diversity beliefs. Results from the supplemental analyses also showed that the effects of DSEL were strongest for male raters. These findings suggest that among individuals who may hold skepticism toward sex-based diversity, exposure to DSEL behaviors is associated with a stronger positive shift in perceptions of female leaders’ competence. DSEL’s emphasis on collaboration, employee development, and change, may effectively counteract pre-existing biases, reducing reliance on stereotypical perceptions, and promoting a reassessment of female leaders’ abilities based on their actual competence (Nishii & Leroy, 2022). Whereas, for individuals with positive sex-based diversity beliefs, DSEL behaviors may reinforce but not significantly alter perceptions since these individuals likely already perceive female leaders as competent. This aligns with research suggesting that individuals with supportive beliefs toward diversity may have already internalized inclusive attitudes, which reduces the incremental impact of diversity-supportive leadership interventions (Plaut et al., 2011).

Finally, in Study 3 we showed that DSEL was both indirectly (via perceptions of female leaders’ competence) and directly associated with a climate for inclusion with the effects being strongest for those with negative sex-based diversity beliefs. These results highlight the potential of DSEL to facilitate attitudinal shifts that contribute to a more inclusive environment. Specifically, when DSEL positively influences perceptions of female leaders’ competence—especially among those with initially negative sex-based diversity beliefs—it creates a ripple effect, enhancing the overall climate for inclusion. This aligns with social cognitive theory, which posits that individual perceptions and attitudes can collectively shape organizational norms and climate (Bandura, 1986a, b; Nishii & Leroy, 2022).

### Theoretical Implications

Our study contributes to the literature in several ways. First, this study offers knowledge about the path from diversity to equity to inclusion (Randel, 2023) by incorporating leadership processes into Bernstein et al.’s (2020) Theory of Generative Interactions organizations. In this way we respond to calls for new theory that can guide ethical action in organizations (Bernstein et al., 2020). Specifically, we advance DSEL as a force that can help overcome exclusionary dynamics (i.e., biased perceptions of female leaders’ competence), and in turn foster more inclusive work environments. Recognizing that “change and learning are endemic to inclusion” (Randel, 2023, p. 13), we argue that, in helping followers overcome challenges and uncertainties (Berger & Calabrese, 1975; Randel, 2023) associated with changing their sex-based biases, DSELs may circumvent backlash, enabling a more accurate understanding of female leaders’ competence (Chang et al., 2019a, b; Day et al., 2022; Dover et al., 2020; Leslie, 2019). This ability of DSEL to tackle followers’ resistance to change is both timely and relevant in the context of changing political climates (e.g., the recent backlash against diversity efforts in corporate America; Elting, 2024).

Second, we contribute to the literature on the unintended consequences of diversity initiatives (Bernstein et al., 2020; Caleo & Heilman, 2019; Dover et al., 2020; Leslie, 2019) and respond to calls for an examination of intervening variables such as diversity beliefs in the path to inclusion (Randel, 2023). This study demonstrated that the effects of DSEL on perceptions of female leaders competence’ were strongest for individuals holding negative sex-based diversity beliefs. These findings can be explained by the added challenges, uncertainties and threats faced by this group, making the effects of DSEL more pronounced (Atewologun et al., 2018; Berger & Calabrese, 1975; Koch et al., 2015; Meyer et al., 2022; Randel, 2023; Tresh et al., 2019). DSELs may provide these individuals with positive developmental experiences that challenges their initial sex-based biases while at the same time reducing backlash by enabling them to continue to feel included through sharing power and encouraging full participation. Research shows that exposure to diversity-supportive behaviors can reduce stereotype-driven evaluations by encouraging individuals to view diverse colleagues through a more equitable and competence-focused lens (Dover et al., 2020; Leslie, 2019; van Knippenberg et al., 2004). In this way, DSEL helps shift the perception of female leaders from being influenced primarily by preconceived biases, to perceptions based on observed abilities. This finding is particularly helpful since existing diversity research has struggled to identify viable strategies for effectively reaching this group of employees (Chang et al., 2019a, b; Dover et al., 2020).

Third, our study adds to the growing body of literature suggesting that target-specific leadership should be a focus of inquiry when addressing change in areas of entrenched beliefs (Barling et al., 2002; Robertson, 2018) such as diversity and inclusion (Bernstein et al., 2020). To date, the limited amount of research linking leadership to diversity outcomes has defaulted to transformational leadership; however, our research investigates assertions by Scheuer and Loughlin (2021) and Lee et al., (2018), regarding the limitations of transformational leadership with regards to diversity contexts. Specifically, we found that general leadership approaches, such as transformational leadership and empowering leadership, may not be sufficiently linked to changing deep-seated biases regarding female leaders. Rather, a *diversity-specific* approach to empowering leadership may be neccessary in overcoming biased perceptions of female leaders’ competence and in turn fostering more inclusive workplaces.

### Practical Implications

Our findings demonstrate that DSELs could enable ethical management practices (Bernstein et al., 2020) by reducing biased perceptions of female leaders’ competence and enabling a more inclusive work environment. Notably, this type of leader may also circumvent backlash that can arise when attempting to institute diversity initiatives (Chang et al., 2019a, b; Dover et al., 2020), particularly on the part of those with negative sex-based diversity beliefs (Byrne et al., 2020; Carlsson & Sinclair, 2020; Christensen & Muhr, 2019; Faniko et al., 2017; Furtado et al., 2021; Gangadharan et al., 2016; Leibbrandt et al., 2018; Nordell, 2021). Further, given that most individuals in hiring positions are men (Chang et al., 2019a, b) and that men also tend to be more biased against female leaders (Atewologun et al., 2018; Bettencourt et al., 2001; Carlsson & Sinclair, 2020; Christensen & Muhr, 2019; Finkelstein et al., 1995; Gangadharan et al., 2016; Gordon & Arvey, 2004; Ibarra & Obodaru, 2008; Koch et al., 2015), there may be strong potential to enact positive change in women’s leadership advancement through DSELs engaging men in these hiring roles as allies (Byrne et al., 2020; Dover et al., 2020; Madsen et al., 2020).

As the business ethics literature suggests, progress on diversity goals is relevant for all stakeholders (Bernstein et al., 2020). In the case of increasing the representation of women in leadership, not only has research suggested that sex-based equity in these roles may have a positive impact on performance outcomes (Galinsky et al., 2011; Meyer et al., 2022; McKinsey & Company, 2015, 2021a, b, c; Moreno-Gómez et al., 2018; Nordell, 2021; ‘The Weaker Sex’, 2015; Tresh et al., 2019), but it has also shown that a greater female presence in leadership has the potential to improve gender stereotypes which also negatively impact men (e.g., men working in traditionally feminine roles such as nursing, health, child or elder care; Galinsky et al., 2011; McKinsey & Company, 2021a; ‘The Weaker Sex’, 2015). Thus, DSEL may serve as one approach organizations can use to challenge sex-based biases and stereotypes without alienating those who can most effectively act as allies (e.g., Byrne et al., 2020), thereby creating a more inclusive workplace for all (Dover et al., 2020).

By demonstrating that DSEL’s influence extends to inclusive work climates via perceptions of female leaders’ competence, this study underscores the importance of embedding DSEL practices into organizations. Encouraging leaders to adopt DSEL behaviors may not only improve individual attitudes toward female leaders, but also foster a climate that values and supports all aspects of diversity. However, organizations must be aware that, while DSEL offers promising avenues for fostering inclusion and advancing sex-based equity in leadership, its implementation may face practical challenges. These challenges may include resistance stemming from entrenched organizational norms, where leadership can often be enacted in more directive than empowering or participative ways. In contrast, DSELs practice facilitation rather than direction of their teams, and focus on skills such as active listening, soliciting diverse viewpoints, and addressing unconscious biases in communication. When it comes to training, organizations can apply exercises and approaches that promote DSEL behaviors such as collaboration and employee development. Role-playing or scenario-based workshops, for instance, can be effective. Here, participants in diverse teams can be presented with specific business challenges to solve. These activities can be complemented by group or individual coaching and feedback sessions to enhance the application of these practices in real-world scenarios.

Training programs often fail because they are not sensitive to participants' reactions to instructors and training methods (Hughes et al., 2016). Hence, through practicing DSELs behaviors such as participative decision-making, facilitators can enhance active trainee engagement, foster mutual understanding, and cultivate shared responsibility for achieving inclusive solutions. Promoting active learning and being able to practice skills in a safe environment can be key to success (Barling et al., 1996), in addition to clear metrics and adequate resources. However, in order to understand which practices are more effective at achieving inclusion goals, leaders should also carefully monitor relevant data, e.g., diversity climate surveys following DSEL training interventions or exposure (Nishii & Leroy, 2022).

Finally, although DSEL was positively related to perceptions of female leaders’ competence and a climate for inclusion, our analyses controlled for prior exposure to female leaders which had a strong relationship with these diversity outcomes. As such, it seems reasonable to consider if organizations ought to *also* focus their efforts on requiring more proportional representation of men and women in leadership roles (e.g., Arnold & Loughlin, 2019; O’Brien & Rickne, 2016). This might involve tracking representation numbers, much like is done with variables such as profitability or safety, in order to increase accountability for diversity outcomes. Drawing upon Bernstein et al.’s (2020) Framework for Inclusive Interactions, having repeated *exposure* to competent female leaders in organizations, over time, will result in the opportunity for individuals to challenge their pre-existing stereotypes and come to recognize female leaders’ abilities. But, as other literature has eloquently argued (e.g., Bernstein et al., 2020; Caleo & Heilman, 2019; Leslie, 2019), one-off efforts in this area often do not have the desired outcomes and can, in fact, create unintended negative outcomes. Incorporating a DSEL approach with other diversity initiatives in a holistic manner may enable sustainable change in the quest for sex-based equity in leadership.

## Limitations and Future Research

Although the models in our study had a strong theoretical foundation (Aguinis & Vandenberg, 2014), giving us confidence in our findings, our cross-sectional design cannot definitively determine causality. The cross-sectional design was selected to provide an initial exploration of the effects of DSEL. This approach is commonly used in early stage research to examine associations and establish foundational insights before pursuing more resource-intensive longitudinal studies (Bryman, 2016; Podsakoff et al., 2003). Further, cross-sample validation is often employed to establish a quasi-longitudinal understanding, as it tests the stability of observed relationships across contexts and sample characteristics (Eden, 2002). By replicating our findings across three studies, along with performing supplemental analyses in two additional samples, we also approximate temporal generalizability as recommended by Dienesch and Liden (1986).

Additional steps were also taken to address concerns of multicollinearity and common method variance. We assessed multicollinearity using Variance Inflation Factors (VIFs) for each predictor variable in the model. We adhered to best practices in management research, which suggest that VIF values below five pose minimal risk for multicollinearity issues (Hair et al., 2014; Kutner et al., 2004). Our VIFs were well below five, ensuring the stability and interpretability of the regression coefficients.

To further verify model stability, we examined condition indices as an additional diagnostic test (Belsley et al., 1980). Consistent with recommended thresholds, condition indices below 30 are considered acceptable for most applied research (Hair et al., 2014). In our model, all condition indices fell well below this threshold, indicating that multicollinearity is unlikely to influence our results significantly. We also incorporated bureaucracy into our analyses to establish divergent validity for DSEL (see Appendix A). This helps to address validity threats due to common method variance (Lindell & Whitney, 2001).

With measurement of DSEL and preliminary relationships between DSEL and diversity outcomes established, future studies that track changes in perceptions of female leaders’ competence and inclusive climates after exposure to DSELs through the use of a longitudinal and/or experimental design would be useful (Antonakis et al., 2010; Bezrukova et al., 2016; Collins & Holton, 2004; Day, 2011; Taris & Kompier, 2003) (see Appendix F for further details). We also recommend that researchers take steps to further refine the measurement and conceptualization of DSEL following best practices in scale development (DeVellis, 2003; Hinkin, 1998) as proposed by Robertson (2018). Conceptually, each of the five components of DSEL are important for circumventing backlash often spurred by diversity initiatives by engaging with and supporting individuals most likely to resist and/or have difficulties with such change efforts (e.g., those with negative sex-based diversity beliefs; Dover et al., 2020). That said, since the five-factor model of DSEL also demonstrated a reasonable fit during CFA, future research may examine whether certain aspects of DSEL are more effective in circumventing backlash and positively influencing perceptions of female leaders’ competence and inclusion. This may be accomplished by separately testing the effects of each of the five components of DSEL through the use of hierarchical regression analysis and/or by testing different combinations of DSEL behaviors through the use of fuzzy-set qualitative comparative analysis (Scheuer et al., 2022).

Future research could also seek to gain a more nuanced understanding of the effects of DSEL by examining additional mediators, such as a measure of backlash or defensiveness based on Leslie (2019), or other exclusionary dynamics (e.g., self-segregation and communication apprehension) found in Bernstein’s et al. (2020) Theory of Generative Interactions. Further exploration into the effects of prior exposure to female leaders is also warranted given the underutilization of intergroup contact theory in management research (Bernstein et al., 2020). It may also be beneficial to explore how individual’s perceptions and beliefs interact with organizational and cultural factors. For example, national societal values related to masculinity, power distance and religiosity activate traditional gender stereotypes (prescribing women as more suitable for supporting roles rather than roles as leaders) and reinforce individuals’ perceptions regarding women’s ability to access leadership positions (Chizema et al., 2015; Grosvold & Brammer, 2011). Future research may delve into these and other contextual factors to better prepare DSELs for tackling the many challenges related to overcoming biased perceptions of female leaders’ competence.

Another avenue for future research would be to include perceptions of female leaders’ communality alongside perceptions of competence. Fiske et al.’s (2002) stereotype content model suggests that out-groups (e.g., women in leadership) perceived as being high in competence can elicit a negative emotional response in the form of envy and resentment, which can lead to unwarranted low perceptions of communal/warmth traits. Meanwhile, there can be a “backlash effect” when women fail to exhibit communal qualities in leadership roles, even when perceived as being highly competent (Ferguson, 2018; Phelan et al., 2008; Rudman & Glick, 2001), which can inhibit their success and advancement in leadership (Leslie, 2019). With their emphasis on collaborative and developmental behaviors along with *showing concern* for others, DSELs are in an ideal position to contribute to less biased perceptions of female leaders’ competence and, perhaps, their communality as well (Nishii & Leroy, 2022). In fact, preliminary evidence from a working study of ours suggests such a relationship could exist.

Finally, although our research focused on improving the perceptions of women in leadership, an examination of other demographics and/or intersectionality in leadership roles could also help promote equality in additional ways. Age (e.g., Scheuer & Loughlin, 2020), sexual orientation (e.g., Clarke & Arnold, 2018; Pellegrini et al., 2020), race (e.g., Hekman et al., 2017), individuals with disabilities, immigrants, low-socioeconomic status individuals (Leslie, 2019), and individuals who embody intersectional identities are additional demographic groups that may be perceived as less competent in leadership roles (Dover et al., 2020). These groups could potentially all benefit from the influence of a DSEL. A key aspect of DSEL is its ability to tailor its approach based on the collective input from its members. Through this adaptive process, DSELs may flexibly enact leadership behaviors based on the needs of the particular context of diversity they are facing (Nishii & Leroy, 2022). With their understanding of multiple biases faced by marginalized groups with intersectional identities and their unique ability to circumvent backlash, DSELs may be effective for mitigating biased perceptions of leadership competence targeted toward these different groups as well.

## Conclusion

Ensuring that women “are elevated to their level of competence—requires more than one top-down structural change. It requires a change of culture” (Nordell, 2021, p. 224). As our study has demonstrated, DSEL has both an indirect (via perceptions of female leaders’ competence) and direct association with a climate for inclusion in the organizational context. Hence, by attenuating sex-based bias (especially from those most predisposed to discriminate against female leaders) and creating a more inclusive environment that all organizational members can benefit from, DSELs shows promise for addressing the wicked policy-performance gap problem of achieving sex-based equity in leadership roles.

## Data Availability

Researchers interested in accessing the data can contact the corresponding author for further information.

## Appendix A: Scale Development Process for Diversity-Specific Empowering Leadership (DSEL)

### Content and Face Validity

In scale development, the primary objective is to create a set of items that clearly represents the content domain (Worthington & Whittaker, 2006). We utilized a deductive method in the development of our DSEL measure by adapting the pre-existing validated Empowering Leadership Questionnaire (Arnold et al., 2000). The five components of general empowering leadership (leading by example, participative decision-making, coaching, showing concern/interacting with the team, and informing) are relevant and can be tailored to be diversity-specific. As such, we modified the existing items to address diversity.

Adapting or modifying existing leadership scales to fit a specific context (in our case diversity) has been a widely used technique in management research (e.g., Aboramadan et al., 2021; Barling et al., 2002; Beauchamp et al., 2010; Conchie & Donald, 2009; Graves et al., 2013; Morton et al., 2011; Robertson, 2018; Robertson & Barling, 2013). The particular steps used for the adaption of these scales differ widely in their complexity and rigor depending on the specific research aims. As our research was meant to be an important *first step* in exploring alternative leadership approaches for fostering less biased and more inclusive environments, our approach was mostly informed by other research at similar stages of the exploration process (e.g., Barling et al., 2002; Graves et al., 2013; Robertson & Barling, 2013). We also incorporated additional steps beyond what was used in this prior work in an effort to enhance the rigor of our research.

Specifically, our approach involved a four-step iterative process. In the first step, we conducted an extensive literature review of the diversity and leadership literatures. From this review, we preliminarily identified leadership approaches and behaviors that were relevant for diverse contexts (such as TL, EL, inclusive leadership, diversity-valuing behavior, transactional leadership, authentic leadership, servant leadership, developmental leadership, change-oriented leadership, and participative/collaborative leadership.).

Next, we conducted in-depth interviews with a purposive sample of senior leaders and their immediate followers, who also occupied leadership roles (*N* = 11, 45% female, age range: 23 to 75 years), from three organizations known for their diversity and inclusion success (Stake, 2005). Using a semi-structured interview protocol, we asked participants to discuss the types of leadership behaviors they felt most positively influenced attitudes and actions toward diversity and inclusion both from a follower and a leader perspective. A template analysis of the data was performed following King and Brooks (2018), whereby codes/themes (in our case leadership behaviors and models) were operationalized a priori based on existing leadership and diversity theory. Subsequently, the initial coding template was modified as new sub-categories were derived inductively from the data. The behaviors associated with empowering leadership, and specifically those associated with Arnold et al.’s (2000) conceptualization of this model, were found to be the most relevant for positively influencing diversity outcomes.

The third step involved the development and refinement of the content and wording of our DSEL scale items. This was a collaborative and iterative process enacted over a period of several months by the members of the research team, each of whom are subject matter expects on the topics of leadership and diversity. As a starting point for this step, we followed the approach utilized by Barling et al. (2002), Graves et al., (2013), and Robertson and Barling (2013). Due to anticipated survey space constraints on subsequent stages of the research we used Srivastava et al.’s (2006) shortened 15-item version of Arnold et al.’s (2000) Empowering Leadership Questionnaire (ELQ) scale (which contains three items from each of the original five components of the model).

Next, we modified these items to ensure that they were appropriate for the diversity context. The specific language used during the modification were informed by the research team’s knowledge on diversity and leadership as well as the findings from the interviews and literature review. The item modifications made based on the interviews and the literature review enhanced the measure’s face validity by aligning item wording and relevance with participants’ real-world experiences and relevant theory. These changes increased the likelihood of accurate interpretation without compromising the theoretical integrity of the constructs being measured.

For the fourth step of the process, we refined the items for clarity, relevance, and interpretation consistency by seeking feedback on the items from several graduate research assistants assigned to support the project, all of which were knowledgeable on the topic of the paper. Based on participant feedback, minor adjustments were made to the items to correct for complex, abstract, or ambiguous wording being used and to ensure that items were relevant across organizational roles and team structures. For example, participants highlighted terms and phrases that needed clarification to better reflect the nuances of empowering leadership behaviors within diverse teams. Participants also found certain terms too formal or broad, which limited their ability to relate directly to real-life applications. By refining items for clarity, relevance, and interpretation, we strengthened the scale’s potential to capture empowering leadership behaviors effectively in diverse organizational contexts.

Through this iterative four-step process of feedback and refinement, we ensured that the final scale items were both clear and applicable, thereby enhancing their validity and reliability for measuring the role of DSEL in supporting diversity and inclusion outcomes.

### Test of Construct Validity

Given that our DSEL scale was adapted from the pre-existing empowering leadership scale developed by Arnold et al. (2000), we conducted a Confirmatory Factor Analysis (CFA) on the Study 1 data (*N* = 236) using Mplus Version 8.6. We tested two models—the original five-factor model as first proposed by Arnold et al. (2000) and a one-factor model [based on Srivastava et al.’s (2006) conceptualization of EL whereby EL was treated as a global measure] given that “each of these models were considered plausible given previous research on empowerment” (Arnold et al., 2000, p. 257). Maximum Likelihood was used to estimate model parameters and the goodness-of-fit of all the CFA models using CFI ≥ 0.95, TLI ≥ 0.95, RMSEA ≤ 0.08 (90% CI ≤ 0.06), and SRMR ≤ 0.08, (Awang, 2012; Brown, 2015; Hu & Bentler, 1999). Results of both the five-factor model (Leading by example—3 items; Participative decision-making—3 items; Coaching—3 items; Showing concern—3 item; Informing—3 items) and the one-factor model exhibited an acceptable fit (CFI = 0.99, TLI = 0.99, RMSEA = 0.09, SRMR = 0.02). The RMSEA value was slightly above the recommended thresholds. However, Kenny et al. (2015) suggested that when employing cut-off values to assess the fit of properly specified models with small degrees of freedom and limited sample sizes, the RMSEA often erroneously suggests a poor fit. In both instances, the chi-square value was also significant. We did anticipate significance because the chi-square index is also affected by sample size whereby a moderate to large sample size will produce statistically significant results (*N* > 200—Jackson et al., 2009; Stride, 2017). See Table 4 for fit indices of the five-factor and one-factor models. Comparisons of the five-factor and one-factor model showed minimal differences; as such, in the interest of parsimony, we proceeded with the one-factor model of DSEL as an overarching measure, which is consistent with other research using the original empowering leadership model (Srivastava et al., 2006).

**Table 4:** Model fit indices for DSEL

	*x* ^*2*^	df	CFI	TLI	RMSEA	SRMR
Five-factor model	239.03*	80	0.99	0.99	0.09	0.02
One-factor model	282.03*	90	0.99	0.99	0.09	0.02

*Indicates significance at the 0.01 level (2-tailed). *N* = 236

To ensure our DSEL construct was distinct from EL, we constructed various CFA models on the DSEL and EL scales using data from Study 1 (*N* = 236) and Study 2 (*N* = 314). See Tables 5 and 6 for the results. An examination of the fit indices for both datasets shows that the two-factor model, in which DSEL and EL are treated as separate factors, fit the data better than a one-factor model, in which DSEL and EL are treated as one overarching leadership measure. Additionally, a chi-squared difference test showed that the two-factor model fit the data better than a one-factor model for both datasets (Δ*χ*^2^ = 750.95, df = 1, *p* < 0.001 and Δ*χ*^2^ = 711.17, df = 1, *p* < 0.001).

**Table 5:** Model fit indices for one and two-factor CFA models for DSEL and EL using Study 1 dataset

	*x* ^*2*^	df	CFI	TLI	RMSEA	SRMR
Two-factor model	1053.11*	404	0.91	0.91	0.08	0.04
One-factor model	1804.03*	405	0.82	0.8	0.12	0.07

*Indicates significance at the *p* < 0.001 level (2-tailed). *N* = 236

**Table 6:** Model fit indices for one and two-factor CFA models for DSEL and EL using Study 2 dataset

	*x* ^*2*^	df	CFI	TLI	RMSEA	SRMR
Two-factor model	1215.17*	404	0.91	0.90	0.08	0.04
One-factor model	1926.34*	405	0.83	0.82	0.1	0.06

*Indicates significance at the *p* < 0.001 level (2-tailed). *N* = 314

### Convergent and Divergent Validity

#### Additional Measures

In addition to the measures included in the main paper for convergent and divergent validity purposes, as well as for our supplemental analyses, we also measured for the following variables:

Transformational Leadership was measured using a 22-item scale by Podsakoff et al. (1996). Sample items include: “Inspires others with his/her plans for the future” and “Shows respect for my personal feelings”. Each item was rated on a five-point scale (1 = Not at all, 5 = Frequently if not always). The internal reliability was an excellent *α* = 0.94.

Transactional Leadership (Contingent Reward) was measured with four items from the MLQ (Avolio & Bass, 2004). Items included: “Expresses satisfaction when I meet expectation” and “Provides me with assistance in exchange for my efforts”. A five-point Likert scale was used (1 = Not at all, 5 = Frequently if not always). The contingent reward subscale showed a good internal reliability *α* = 0.81.

Leader diversity-valuing behavior was measured using a three-item scale by Hekman et al. (2017). Sample items include: “Values working with a diverse group of people” and “Understands and respects cultural, religious, gender, and racial differences”. Each item was rated on a five-point scale (1 = Not at all, 5 = Frequently if not always). The internal reliability was an excellent *α* = 0.90.

Inclusive leadership was measured with a nine-item scale by Carmeli et al. (2010). Sample items included: “Is open to hearing new ideas” and “Is ready to listen to my requests”. A five-point Likert scale was used (1 = Not at all, 5 = To a large extent). The scaled showed an excellent internal reliability *α* = 0.95.

Bureaucracy was also included to offer evidence of divergent validity for the DSEL measure and to determine if Common Method Variance (CMV) was operative. Bureaucracy assesses the relative emphasis on rules and “red tape” within organizations (Rafferty & Griffin, 2004). Three items were used to measure this construct based on Hage and Aiken (1967) and Rafferty and Griffin (2004). One such item in this scale was “Decisions must go through many levels of management before they are finalized.” The internal reliability for this scale was acceptable, *α* = 0.72.

### Results

To determine how DSEL was related to other leader behavior instruments, we conducted simple bivariate correlation analyses with empowering leadership, transformational leadership, and transactional leadership (contingent reward), as these are all common constructs that consider the leader/follower relationship (e.g., full-range leadership model—Avolio & Bass, 2004), and also with two established diversity-focused leadership measures, leader diversity-valuing behavior (Hekman et al., 2017) and inclusive leadership (Carmeli et al., 2010), using the Study 1 dataset (*N* = 236) and an additional dataset (*N* = 313), We also included a measure of bureaucracy (*α* = 0.72; Hage & Aiken, 1967) to establish divergent validity.

Bureaucracy was chosen to assess divergent validity since other research has shown this measure to be theoretically unrelated to other positive leadership models that are relevant to diversity contexts and that are also conceptually similar to DSEL such as transformational leadership and leader gender-based allyship (Lyubykh et al., 2023; Rafferty & Griffin, 2004, 2006). Therefore, we expected a similar relationship to occur with our DSEL measure. That being said, from a theoretical standpoint bureaucracy refers to organizational structures characterized by rigid hierarchies, standardized procedures, and formalized rules (Mintzberg, 1979). Research has shown that bureaucratic environments can constrain the effectiveness of empowering leadership by limiting leaders' autonomy and flexibility to implement inclusive and supportive practices (Parker, 2014). In highly bureaucratic organizations, DSEL behaviors may be less impactful due to structural limitations, which can restrict leaders’ capacity to foster inclusive and empowering environments. Hence, as we noted in our manuscript, we decided to control for bureaucracy in order to account for these structural constraints, ensuring that the observed effects of DSEL on leadership perceptions were not confounded by organizational rigidity. However, bureaucracy was not correlated with DSEL and the substantive results were not altered when it was included or excluded in our analyses,. As such, in the interest of parsimony, we excluded this variable from the final models/analyses presented in our paper. Tables 7 and 8 present the scale means, standard deviations, and bivariate correlations between DSEL and comparison instruments.

**Table 7:** Overall bivariate correlations between DSEL and leader/follower and bureaucracy comparison instruments

Scale	*M*	SD	1	2	3	4	5
1. Diversity-specific empowering leadership (DSEL)	3.66	1.04	1	0.81**	0.73**	0.70**	0.12
2. Empowering leadership	3.91	0.91		1	0.82**	0.80**	0.11
3. Transformational leadership	3.78	0.74			1	0.69**	0.90
4. Transactional leadership	3.64	0.93				1	0.16*
5. Bureaucracy	3.85	0.88					1

*Correlation is significant at the 0.05 level (2-tailed)

**Correlation is significant at the 0.01 level (2-tailed). *N* = 236

**Table 8:** Overall bivariate correlations between DSEL and diversity-focused leadership comparison instruments

Scale	*M*	SD	1	2	3
1. Diversity-specific empowering leadership (DSEL)	3.67	1.02	1	0.76**	0.80**
2. Leader diversity-valuing behavior	4.02	0.98		1	0.68**
3. Inclusive leadership	4.01	0.89			1

*Correlation is significant at the 0.01 level (2-tailed)

**Correlation is significant at the 0.01 level (2-tailed). *N* = 236

As predicted, the correlations between DSEL, empowering leadership (*r* = 0.807, *p* < 0.01), transformational leadership (*r* = 0.730, *p* < 0.01), transactional leadership (contingent reward; *r* = 0.698, *p* < 0.01), leader diversity-valuing behavior (*r* = 0.76, *p* < 0.01), and inclusive leadership (*r* = 0.80, *p* < 0.01) were all significant and in the positive direction. Additionally, bureaucracy and DSEL showed no significant relationship (*r* = 0.124, *p* = n.s) as expected.

In addition to using bureaucracy for divergent validity purposes, we also incorporated bureaucracy into the various regression models for Hypotheses 1–3 to ensure the observed effects of DSEL on leadership perceptions were not confounded by organizational rigidity (Mintzberg, 1979; Parker, 2014) (see Appendix A). Bureaucracy displayed non-significant relationships with the substantive variables, providing additional evidence that the influence of common method variance was not likely a concern in our study.

We also tested various CFA models with DSEL and the other alternative leadership constructs considered in the study in order to provide additional evidence of its distinctiveness to these models. To ensure that our DSEL construct was distinct from Transformational Leadership (TL), we conducted confirmatory factor analyses (CFA) on the DSEL and TL scales using data from Study 1 (*N* = 236). See Table 9 for the results. An examination of the fit indices shows that the DSEL only model, in which DSEL is treated as a single factor, fit the data better than the DSEL + TL model, where DSEL and TL are combined into a two-factor model.

The DSEL only model demonstrated strong fit indices (*χ*^2^(90) = 290, *p* < 0.001, CFI = 0.939, TLI = 0.929, RMSEA = 0.0971, SRMR = 0.0352), while the DSEL + TL model exhibited poorer fit (*χ*^2^(629) = 1984, *p* < 0.001, CFI = 0.826, TLI = 0.815, RMSEA = 0.0955, SRMR = 0.0636). A chi-square difference test (Δ*χ*^2^ = 1694, Δdf = 539, *p* < 0.001) suggests a significant difference between the "DSEL only" and "DSEL + TL" models, indicating that the DSEL only model provided a better fit to the data than the DSEL + TL model.

**Table 9:** Model fit indices for one and two-factor CFA models for DSEL and Transformational Leadership using Study 1 dataset

Model	*x* ^*2*^	df	CFI	TLI	RMSEA	SRMR
Two-factor model	1984*	629	0.826	0.815	0.0955	0.0636
One-factor model	290*	90	0.939	0.929	0.0971	0.0352

*Indicates significance at the *p* < 0.001 level (2-tailed). *N* = 236

To assess the distinctiveness of the DSEL construct from Diversity-Valuing Behavior (DVB), confirmatory factor analyses (CFA) were conducted on the DSEL and DVB scales using data from the Study 3 (*N* = 313). See Table 10 for the results. The fit indiced indicate that the DSEL only model, treating DSEL as a single factor, fit the data better than the DSEL + DVB model, in which DSEL and DVB were combined into a two-factor model.

The DSEL only model demonstrated good fit indices (*χ*^2^(90) = 589, *p* < 0.001, CFI = 0.905, TLI = 0.889, RMSEA = 0.133, SRMR = 0.0449), while the DSEL + DVB model showed a poorer fit (*χ*^2^(135) = 912, *p* < 0.001, CFI = 0.87, TLI = 0.86, RMSEA = 0.14, SRMR = 0.05). A chi-square difference test revealed a significant difference between the two models (Δ*χ*^2^ = 323, Δdf = 45, *p* < 0.001), indicating that the DSEL only model provided a better fit than the DSEL + DVB model.

**Table 10:** Model fit indices for one and two-factor CFA models for DSEL and diversity-valuing behavior using Study 3 dataset

Model	*x* ^*2*^	df	CFI	TLI	RMSEA	SRMR
Two-factor model	912*	135	0.87	0.86	0.14	0.05
One-factor model	589*	90	0.905	0.889	0.133	0.0449

*Indicates significance at the *p* < 0.001 level (2-tailed). *N* = 313

To evaluate whether the DSEL construct was distinct from Inclusive Leadership, confirmatory factor analyses (CFA) were conducted on the DSEL and Inclusive Leadership scales using data from the Study 3 (*N* = 313). See Table 11 for the results. The fit indices suggested that the DSEL only model, which treats DSEL as a single factor, fit the data better than the DSEL + Inclusive Leadership model, where DSEL and Inclusive Leadership were combined into a two-factor model.

The DSEL only model showed good fit indices (*χ*^2^(90) = 589, *p* < 0.001, CFI = 0.905, TLI = 0.889, RMSEA = 0.133, SRMR = 0.0449), while the DSEL + Inclusive Leadership model demonstrated poorer fit (*χ*^2^(252) = 1659, *p* < 0.001, CFI = 0.827, TLI = 0.811, RMSEA = 0.134, SRMR = 0.0663). A chi-square difference test revealed a significant difference between the two models (Δ*χ*^2^ = 1070, Δdf = 162, *p* < 0.001), indicating that the DSEL only model provided a better fit than the DSEL + Inclusive Leadership model.

**Table 11:** Model fit indices for one and two-factor CFA models for DSEL and inclusive leadership using Study 3 dataset

Model	*x* ^*2*^	df	CFI	TLI	RMSEA	SRMR
Two-factor model	1659*	252	0.827	0.811	0.134	0.0663
One-factor model	589*	90	0.905	0.889	0.133	0.0449

*Indicates significance at the *p* < 0.001 level (2-tailed). *N* = 313

## Appendix B: Diversity-Specific Empowering Leadership (DSEL) Scale

**Table:**

Sub-dimension	Original	Modified for diversity-specific
Leads by example	Sets high standards for performance by his/her own behavior	Sets high standards for our team’s diversity and inclusion efforts by his/her own behavior
Leads by example	Leads by example	Leads by example when it comes to diversity and inclusion
Leads by example	Sets a good example by the way he/she behaves	Sets a good example when it comes to diversity and inclusion through his/her behaviors
Participative decision-making	Encourages team members to express ideas/suggestions	Encourages team members to express ideas/suggestions pertaining to diversity and inclusion
Participative decision-making	Uses suggestions of other managers to make decisions that affect us	Uses team members’ suggestions to make decisions on diversity and inclusion
Participative decision-making	Gives all team members a chance to voice their opinions	Gives all team members a chance to voice their opinions regarding diversity and inclusion
Coaching	Helps team members identify areas where they need more training	Helps team members identify areas where they need more training as it pertains to diversity and inclusion
Coaching	Teaches our team members how to solve problems on our own	Teaches our team members how to solve diversity and inclusion problems on our own
Coaching	Supports the efforts of our team	Supports our team’s diversity and inclusion efforts
Informing	Explains company goals to team members	Explains company goals related to diversity and inclusion to team members
Informing	Explains the purpose of the company's policies to team members	Explains the purpose of the company's policies pertaining to diversity and inclusion to team members
Informing	Explains rules and expectations to team members	Explains rules and expectations regarding diversity and inclusion to team members
Show concern/interact with team	Shows concern for team members' well-being	Shows concern for team members’ unique and diverse wellbeing needs
Show concern/interact with team	Patiently discusses team members' concerns	Patiently discusses team members' concerns regarding diversity and inclusion
Show concern/interact with team	Shows interest in team members' success	Shows interest in the success of our team’s diversity and inclusion efforts

The ‘original’ empowering leadership items were taken from Srivastava et al., (2006), which was a shortened version of Arnold et al.’s (2000) empowering leadership questionnaire. Following Barling et al. (2002), each of the items were modified to address the diversity-specific context of the study. The stem for each item was the following: “please rate your manager/supervisor on the extent to which he/she performs the following behaviors…” and each item was measured on a 1—strongly disagree to 5—strongly agree scale.

## Appendix C: Supplemental analysis to Study 1

Using a separate sample (*N* = 510), we developed a diversity-specific transformational leadership (DSTL) measure (*α* = 0.96) following the same procedures used for DSEL (See Appendix A) and then tested the effects of DSTL on perceptions of female leaders’ competence. The effect of DSTL was not significant (*β* = 0.098, *p* = n.s).

We also ran various regression models using the Study 1 sample (*N* = 236) whereby we tested the effects of DSEL with and without alternative leadership models (transactional leadership, EL, TL, and DSTL). Transactional leadership (*β* = 0.11, *p* = n.s), empowering leadership (*β* = 0.16, *p* = n.s), transformational leadership (*β* = 0.11, *p* = n.s) and DSTL (*β* = − 0.009, *p* = n.s) were not statistically significant in predicting perceptions of female leaders’ competence alongside DSEL. Whereas the effects of DSEL on perceptions of female leaders’ competence were statistically significant. Specifically, when compared to transactional leadership, empowering leadership, transformational leadership, and DSTL, respectively, DSEL explained variance (*β* = 0.25, Δ*R*^2^ = 0.03*, p* < 0.001; *β* = 0.20, Δ*R*^2^ = 0.01*, p* = 0.03; *β* = 0.26, Δ*R*^2^ = 0.03*, p* = 0.002; *β* = 0.34, Δ*R*^2^ = 0.02*, p* = 0.02) above and beyond each of these other leadership constructs.

Using a fourth sample (*N* = 313), we tested the effects of leader diversity-valuing behavior (Hekman et al., 2017) and inclusive leadership (Carmeli et al., 2010) on perceptions of female leaders’ competence and a climate for inclusion. DSEL (*β* = 0.21, *p* = 0.004; *β* = 0.18, *p* = 0.02) was a stronger predictor of perceptions of female leaders’ competence than both diversity-valuing behavior (*β* = 0.05, *p* = n.s) and inclusive leadership (*β* = 0.07, *p* = n.s), and also explained variance (Δ*R*^2^ = 0.05*, p* < 0.001; Δ*R*^2^ = 0.04*, p* < 0.001) above and beyond these other leadership constructs. DSEL (*β* = 0.72,* p* < 0.001; *β* = 0.48,* p* < 0.001; *β* = 0.47,* p* < 0.001) was also a stronger predictor of a climate for inclusion than diversity-valuing behavior (*β* = − 0.05, *p* = n.s) and inclusive leadership (*β* = 0.26,* p* < 0.001), and also explained variance (Δ*R*^2^ = 0.21*, p* < 0.001; Δ*R*^2^ = 0.08*, p* < 0.001) above and beyond these other leadership constructs.

These results yielded mostly moderate to small effect sizes. As several researchers have noted that even small effects can have practical importance (e.g., Abelson, 1985; Eagly & Carli, 2003; Nordell, 2021; Purvanova & Muros, 2010; Scheuer & Loughlin, 2020). In our case, although the effects of DSEL were modest in some cases, DSEL still offers an important step forward toward reducing sex-based bias and enabling inclusivity.

We also tested various CFA models with DSEL and these other leadership constructs. In each case, the best fitting model was the one in which DSEL was treated as its own distinct factor (see Appendix A). Based on the supplemental analyses results, and in the interest of parsimony, we proceeded with our measure of DSEL to test our subsequent hypotheses.

To further justify the inclusion of our control variables, we conducted preliminary analyses to examine their relationships with our dependent variables. We used correlation and regression analyses to assess each control’s impact on our outcomes. Results indicated that rater and leader sex and prior exposure to female leaders were significantly correlated/associated with our outcomes. For example, participant sex showed a significant negative association/correlation and leader sex and prior positive exposure to female leaders showed a significant positive association/correlation with our outcomes. These significant associations reinforced the theoretical rationale for including these controls in this study and subsequent studies to enhance the accuracy and validity of our model.

## Appendix D: Supplemental analysis to Study 2

We conducted two supplemental moderation analyses, one with rater sex as a moderator (when controlling for leader sex) and the other with leader sex as a moderator (when controlling for rater sex) in the relationship between DSEL and perceptions of female leaders’ competence. Although the overall moderation coefficients were non-significant (*b* = 0.068, *p* = 0.149, *b* = 0.022, *p* = 0.697), when considering the simple slopes, a stronger effect was observed for the male raters (*b* = 0.310, *p* < 0.001) versus female raters (*b* = 0.174, *p* = 0.05, *R*^2^ = 0.43), and for male leaders (*b* = 0.264, *p* = 0.003) versus female leaders (*b* = 0.219, *p* = 0.02, *R*^2^ = 0.16), suggesting DSEL may have more resounding effects on perceptions of female leaders’ competence for male raters and male leaders. This may be an interesting observation for future inquiry.

## Appendix E: Supplemental analysis to Study 3

In recognizing that eliminating bias and discrimination is considered to be a precondition for inclusion experiences (Nishii & Leroy, 2022), we treated perceptions of female leaders’ competence as a mediator in the relationship between DSEL and a climate for inclusion. That said, while the constructs in our paper have been treated in this manner (since that has been how they have been conceptualized in the literature; Randel, 2023), alternative pathways are certainly still plausible. For instance, a favorable climate for inclusion may attenuate biased perceptions of female leaders’ competence (Caleo & Heilman, 2019; Nishii, 2013).

This alternative model may be explained by Uncertainty Reduction Theory (URT; Berger & Calabrese, 1975). URT asserts that people by nature strive to minimize uncertainty and enhance predictability about the behavior of others through proactive engagement or by building on past interactions. DSELs believe in collaborative interactions with followers (Nishii & Leroy, 2022). They provide opportunities for their followers to actively participate in change and can encourage their followers to challenge traditional ideas and actions (Kotter, 1995). In the context of diversity, this could involve coaching followers on identifying and countering biases in their day-to-day workgroup activities or processes (Nishii & Leroy, 2022). URT also provides cues in terms of the anticipated pathways. Climate for inclusion provides such cues in terms of explaining mechanism of the relationship between DSEL and followers’ perceptions of female leaders’ competence. Consistent with tenets of URT, we argue that DSEL with its focus on empowerment and collaboration in decision-making—a critical factor in the emergence of climate for inclusion (Shore et al., 2011) can positively enhance followers’ perceptions of female leaders’ competence. DSELs with their ability to enhance intrinsic motivation (i.e., create psychological empowerment) can contribute to the create of a climate for inclusion necessary to satisfy followers needs related to competence, belongingness and autonomy (Nishii & Leroy, 2022). Therefore, DSELs can have a positive effect on perceptions of female leaders’ competence via positive climate for inclusion.

It may also be plausible that both climate for inclusion and perceptions of female leaders’ competence act as mutually reinforcing mechanisms to the path to inclusion via a feedback loop (Randel, 2023). This feedback loop aligns with the social identity theory framework, as outlined by Jenkins (2004), who described social identity as a dynamic and ongoing process shaped by situationally defined social interactions over time. Bernstein et al. (2020) offer a clear example of how outcomes may link back to inputs within the inclusion literature. Their work highlights a feedback loop in which inclusion enhances both the willingness and ability to engage with diverse individuals. They argue that motivation and capability to connect with diverse others not only shape perceptions of inclusion but are also reinforced—or diminished—by the level of inclusion present. Despite this insight, as Randel (2023) points out, the feedback loop remains underdeveloped in the inclusion literature, making it a promising area for future research.

Our empirical findings offer some support for these alternative pathways whereby a climate for inclusion was found to mediate the relationship between DSEL and perceptions of female leaders’ competence and perceptions of female leaders’ competence was also found to mediate the relationship between DSEL and climate for inclusion. Specifically, the mediating effects of climate for inclusion on the relationship between DSEL and perceptions of female leaders’ competence was significant, *b* = 0.094, SE = 0.032, 95% CI (0.03, 0.055), *R*^2^ = 0.42 (perceptions of female leader’s competence), *R*^2^ = 0.53 (climate for inclusion). Likewise, the indirect (mediated) effect of DSEL on climate for inclusion via perceptions of female leaders’ competence was also statistically significant, *b* = 0.04, SE = 0.02, 95% CI (0.004, 0.08), *R*^2^ = 0.52. However, such effects were found to hold true regardless of individual’s level of sex-based diversity beliefs. The moderating effects of sex-based diversity beliefs on the relationship between DSEL and climate for inclusion was not statistically significant (*b* = 0.011, *p* = 0.813). Nor were the indirect (mediated) effects of the interaction of DSEL with sex-based diversity beliefs via climate for inclusion on perceptions of female leaders’ competence, *b* = 0.002, SE = 0.008, 95% CI (− 0.014, 0.019). These results prompted our decision to retain perceptions of female leaders’ competence as our mediator in the final model, since understanding how to impact attitudes and behaviors among those most resistant to change (i.e., those with negative sex-based diversity beliefs) was a primary aim of our study. Further, since a climate for inclusion is likely to be a longer-term effect relative to perceptions of female leaders’ competence (which may be more proximal in its association with leader behavior), it was more appropriate for a climate for inclusion to be positioned at the end of the model.

In either case DSEL still serves as the main driver of the process. Hence DSEL was treated as our main predictor. As discussed in the manuscript, the critical aspect of DSEL in initiating the path to inclusion can be explained with respect to its effects on backlash. In general, workplace backlash is considered as direct or indirect forms of resistance toward diversity efforts for a more inclusive workplace (Lee, 2023). Most backlash happens because of the individual’s desire to maintain the group-based hierarchy prevalent in society (Lee, 2023). The unique impact of DSEL’s collaborative behaviors on uncertainty reduction (Berger & Calabrese, 1975; Randel, 2023) allows these leaders to challenge social norms prevalent in society related to female leaders’ competence. Additionally, the coaching component helps followers correct stereotypes and assumptions they may hold. This is especially relevant for people with negative sex-based diversity beliefs due to the added challenges and threats faced by this group. Overall, our study shows the powerful influence of DSELs when tackling followers’ resistance to change. This finding is both timely and relevant in the context of changing political climate in USA and the recent backlash against diversity efforts in the corporate America (Elting, 2024).

## Appendix F: Guidance on longitudinal designs and interventions

As recommended by Taris and Kompier (2003), longitudinal designs are essential for examining changes over time and making causal inferences in leadership research. Therefore, in future studies, we propose implementing a longitudinal design to assess changes in perceptions of female leaders at multiple intervals following DSEL interventions or exposure. In their Meta-Analytical Integration of Over 40 Years of Research on Diversity Training Evaluation Bezrukova et al. (2016), found that the time between the end of diversity training and outcome measures ranged from immediately after training to 24 months (*x* = 0.83, SD = 3.09). Further, according to Bezrukova et al. (2016), long-term effects of diversity training mainly result from more permanent changes in beliefs, expectations, scripts, attitudes, and other factors. Long-term evaluation typically occurs after some time lag (ranges from 1 month to 4 years, M 7.23 months, SD 5.49 in their data).

Based on this information and following the model of longitudinal studies in leadership development research (Day, 2011), we suggest for survey data to be collected from employees on perceptions of female leaders’ competence in the following increments following DSEL interventions or exposure (baseline, 1 month, 6 months, 1 year, and 2 years).

It could also be interesting to assess changes in a climate for inclusion and perhaps also in sex-based diversity beliefs over time following a DSEL intervention. While sex-based diversity beliefs may be more difficult to change than one’s immediate perceptions of female leaders’ competence, it may be possible that both may change with long enough exposure to a DSEL.

In this case, additional data collection may be needed (approximately 1–2 months prior to and following the above proposed time increments) to account for these variables and/or a staggered data collection may be used whereby only certain variables are collected at each time period.

Based on our conceptual model, we recommended the following for the order in which data collection and/or analysis should initially occur for each of the variables in the model: sex-based diversity beliefs should come first in the sequence ideally prior to DSEL intervention and/or exposure (since DSEL is expected to differently impact individuals who have negative sex-based diversity beliefs at the onset). This would be followed by DSEL (as our main predictor), perceptions of female leaders’ competence (as a mediator), and then finally climate for inclusion should be last in the sequence (as our final outcome).

By capturing data at these time points, we could investigate how DSEL behaviors influence perceptions and inclusive climates over time and whether such changes are sustainable in the long-term (Bezrukova et al., 2016), following the model of longitudinal studies in leadership development research (Day, 2011). Furthermore, this approach could allow for exploring intermediate outcomes, such as backlash (Leslie, 2019), that may serve as additional mediators in the relationship between DSEL, perceptions of female leaders’ competence, and climate for inclusion, providing a more nuanced understanding of DSEL’s impact.

Experimental research designs offer a powerful means to infer causality by manipulating the independent variable and controlling for extraneous factors. In line with the call from Antonakis et al. (2010) for more experimental studies in leadership research, we propose a randomized controlled trial (RCT) as another future research strategy. By introducing a DSEL intervention (e.g., training) in a controlled setting and measuring changes in perceptions of female leaders and perhaps also a climate for inclusion and sex-based diversity beliefs over time, we could directly test DSEL’s causal impact. This approach would allow us to address the limitations of the cross-sectional design while contributing more robust evidence of DSEL’s influence on diversity outcomes. Additionally, RCTs could capture the sustained effects of DSEL by comparing experimental and control groups at multiple post-intervention intervals, as recommended in leadership intervention studies (Collins & Holton, 2004).

Interventions on reducing negative sex-based diversity beliefs should focus on change strategies to reduce direct and indirect bias against women, stereotyping, and the adverse effect of career systems (Kossek et al., 2017). Examples (other than DSELs) include enhancing the representation of women in leadership positions; using cluster hiring to minimize tokenism and stigmatization; objective and transparent HRM practices; leadership development advancing equality in selection and evaluation; transparency in evaluation and rewards; and implementing female friendly work-life support programs or policies.

Meta-analytic evidence (e.g., Halliday et al., 2021) suggests that the presence of women in leadership ranks is even more salient in contexts where there is greater bias based on sex (less sex-based equality). Considering the macro-level findings of the powerful effect of the presence of women in organizational leadership positions, we expect that interventions focused on enhancing representation of women in upper echelons of management will mitigate individuals’ negative sex-based diversity beliefs and enhance their perceptions about women’s leadership competence. Specifically, the factors such as openness to diversity, leadership support for women (e.g., DSEL behaviors such as coaching, networking and mentoring), and work-life balance programs can enhance employee perceptions of organizational support for the career progression for women. This could contribute reversing members’ negative sex-based diversity beliefs.
